# Tyrosyl phosphorylation of KRAS stalls GTPase cycle via alteration of switch I and II conformation

**DOI:** 10.1038/s41467-018-08115-8

**Published:** 2019-01-15

**Authors:** Yoshihito Kano, Teklab Gebregiworgis, Christopher B. Marshall, Nikolina Radulovich, Betty P. K. Poon, Jonathan St-Germain, Jonathan D. Cook, Ivette Valencia-Sama, Benjamin M. M. Grant, Silvia Gabriela Herrera, Jinmin Miao, Brian Raught, Meredith S. Irwin, Jeffrey E. Lee, Jen Jen Yeh, Zhong-Yin Zhang, Ming-Sound Tsao, Mitsuhiko Ikura, Michael Ohh

**Affiliations:** 1 Department of Laboratory Medicine and Pathobiology, University of Toronto, 661 University Avenue, Toronto, ON M5G 1M1 Canada; 2 Department of Biochemistry, University of Toronto, 661 University Avenue, Toronto, ON M5G 1M1 Canada; 3 Princess Margaret Cancer Centre, University Health Network and Department of Medical Biophysics, University of Toronto, 101 College Street, Toronto, ON M5G 1L7 Canada; 4 Princess Margaret Cancer Centre, University Health Network and Department of Pathology, University of Toronto, Toronto, ON M5G 1L7 Canada; 5 Peter Gilgan Centre for Research and Learning, The Hospital for Sick Children, 686 Bay Street, Toronto, ON 5G OA4 Canada; 6 Lineberger Comprehensive Cancer Center, University of North Carolina, Chapel Hill, NC 27599 USA; 7 Department of Medicinal Chemistry and Molecular Pharmacology, Center for Cancer Research and Institute for Drug Discovery, Purdue University, 720 Clinic Drive, West Lafayette, IN 47907 USA; 8 Department of Surgery, University of North Carolina, Chapel Hill, NC 27599 USA; 9 Department of Pharmacology, University of North Carolina, Chapel Hill, NC 27599 USA

## Abstract

Deregulation of the RAS GTPase cycle due to mutations in the three *RAS* genes is commonly associated with cancer development. Protein tyrosine phosphatase SHP2 promotes RAF-to-MAPK signaling pathway and is an essential factor in RAS-driven oncogenesis. Despite the emergence of SHP2 inhibitors for the treatment of cancers harbouring mutant KRAS, the mechanism underlying SHP2 activation of KRAS signaling remains unclear. Here we report tyrosyl-phosphorylation of endogenous RAS and demonstrate that KRAS phosphorylation via Src on Tyr32 and Tyr64 alters the conformation of switch I and II regions, which stalls multiple steps of the GTPase cycle and impairs binding to effectors. In contrast, SHP2 dephosphorylates KRAS, a process that is required to maintain dynamic canonical KRAS GTPase cycle. Notably, Src- and SHP2-mediated regulation of KRAS activity extends to oncogenic KRAS and the inhibition of SHP2 disrupts the phosphorylation cycle, shifting the equilibrium of the GTPase cycle towards the stalled ‘dark state’.

## Abstract (web summary)

Deregulation of the RAS GTPase cycle due to mutations in *RAS* genes is commonly associated with cancer development. Here authors use NMR and mass spectrometry to shows that KRAS phosphorylation via Src alters the conformation of switch I and II regions and thereby impacts the GTPase cycle.

## Introduction

Deregulation of the RAS GTPase cycle due to mutations in *RAS* is commonly associated with cancer initiation and progression and several developmental syndromes, referred to as “RASopathies”^1^. While there are three human *RAS* genes (*HRAS*, *NRAS*, and *KRAS*) that encode highly related 188–189 amino acid proteins, *KRAS* is the most frequently mutated oncogene in human cancers, accounting for up to 25% of lung, 40% of colorectal, and 95% of pancreatic cancers^2^. RAS is a small GTPase protein that cycles between GDP-loaded inactive and GTP-loaded activated forms, which adopt distinct conformations at switch I (residues 30–38) and switch II (59–72) regions near the nucleotide-binding site^3^. RAS is activated by guanine nucleotide-exchange factors (GEFs) and the GTP-bound form binds and activates effector proteins, such as RAF. RAS activation is terminated by hydrolysis of GTP, which is accelerated by GTPase-activating proteins (GAPs); however, this step of the GTPase cycle is impaired by most oncogenic RAS mutations^4^.

We previously showed that c-Src (henceforth referred to as Src) binds to and phosphorylates H/NRAS, which was associated with RAF displacement and the attenuation of downstream mitogen-activated extracellular signal-regulated kinase (MEK)-to-extracellular signal–regulated kinase (ERK) and phosphoinositide-3 kinase-to-AKT signaling^5^. Conversely tyrosyl-phosphorylated H/NRAS (pH/NRAS) can be dephosphorylated by SHP2 protein tyrosine phosphatase (PTP), which restores H/NRAS binding to RAF and reactivates downstream signaling^6^. We further showed that pharmacologic inhibition of SHP2 activity attenuates the progression of spontaneous glioblastoma in a mutant HRAS knock-in glioma mouse model^6^. These results taken together support the notion that one of the SHP2 functions is as a direct activator of RAS. Subsequently, a recent series of reports have demonstrated that inhibition of SHP2 suppresses the growth of mutant KRAS-driven lung cancer^7^ and pancreatic ductal adenocarcinoma (PDAC)^8,9^, as well as gastroesophageal cancer with amplification of otherwise wild-type (WT) KRAS^10^. Another proposed model is that inhibition of SHP2 disrupts GTP loading of RAS by the GEF Son of Sevenless 1 (SOS1), suggesting that SHP2 functions by coordinating adaptor proteins on the cell membrane^11^. However, it remains unclear how SHP2 precisely regulates KRAS-to-mitogen-activated protein kinase (MAPK) pathway since oncogenic KRAS mutations confer resistance to GAPs and most would therefore exhibit elevated GTP loading even in the absence of SOS activity^4,12^.

Here, using real-time nuclear magnetic resonance (NMR) and mass spectrometry (MS), we show definitively that KRAS is phosphorylated via Src on Tyr32 and Tyr64, which alters the conformation of switch I and II regions, respectively, negatively impacting every step of the GTPase cycle. We show specifically that tyrosyl phosphorylation of KRAS markedly attenuates its sensitivity to the activities of GEF and GAP and profoundly impairs its binding affinity to the effector RAF. Intrinsic nucleotide exchange was however enhanced, thus GTP-loaded phosphorylated KRAS (pKRAS) accumulated in a dark, “ready-to-serve,” state that can be rapidly unleashed via dephosphorylation by SHP2 PTP. Notably, common oncogenic KRAS mutants such as G12V and G12D were not recalcitrant to phosphorylation-mediated regulation, and pharmacologic inhibition of SHP2 led to the accumulation of silenced pKRAS, supporting the potential clinical utility of manipulating the Src- and SHP2-mediated phosphorylation cycle of KRAS in the management of KRAS-driven cancers.

## Results

### Pharmacologic inhibition of SHP2 reduces PDAC tumor growth

*KRAS* mutations are detected in >90% of PDAC, one of the deadliest cancers without cure or effective treatment^13^, thus a highly relevant cancer type to evaluate the therapeutic utility of SHP2 inhibitors. Here we investigated the effectiveness of a next-generation, cell-permeable catalytic inhibitor of SHP2, 11a-1^14^. Notably, a panel of PDAC cell lines, including those harboring the most frequent mutations G12V or G12D as well as PDAC patient-derived xenograft (PDX) cells were sensitive to 11a-1 treatment (Supplementary Fig. 1a, b), which attenuated the level of epidermal growth factor (EGF)-induced pSHP2, pERK, and pAKT (Supplementary Fig. 1c) while increasing the level of cleaved PARP and caspase 9 in a dose-dependent manner (Supplementary Fig. 1d). In addition, molecular inhibition of SHP2 via CRISPR/Cas9-mediated knockout of *SHP2* in the PDAC cell line CFPAC1 harboring the *KRAS*^*G12V*^ mutation markedly attenuated its rate of cell proliferation (Supplementary Fig. 1e). Moreover, PDAC cell lines grown in three-dimensional (3D) culture or as organoids exhibited dose-dependent sensitivity to 11a-1 as well as an allosteric SHP2 inhibitor SHP099^15^ (Supplementary Fig. 1f, g, h).

We next interrogated the antitumor efficacy of SHP099 in vivo. We first determined the maximum tolerable dose of SHP099 in SCID mice as only nude mice were used in a previous report^15^. We observed no discernable weight loss in 100 mg/kg treatment group while 150 mg/kg treatment showed slight weight loss and 200 and 250 mg/kg treatments were intolerable (Supplementary Fig. 2a, b). SCID mice bearing subcutaneous PDAC xenografts derived from PDX cells OCIP.343 harboring *KRAS*^*G12D*^ were treated with vehicle or SHP099 at 100 mg/kg as well as the MEK inhibitor Trametinib as a positive control, which was previously determined to have a significant antitumor effect in a similar PDX model (Supplementary Fig. 2c). SHP099 treatment resulted in a significant tumor growth inhibition similar to Trametinib compared to vehicle (Supplementary Fig. 3a). Treatment with SHP099 was also well tolerated with no obvious weight loss over the course of treatment (Supplementary Fig. 3b). These observations were associated with reduced levels of pERK, pMEK, and pAKT while the level of cleaved Caspase-3 was increased in the treatment group (Supplementary Fig. 3c, d). Consistent with recent reports^7–11^, these results support the potential utility of SHP2 inhibitors in the treatment of mutant KRAS-driven PDAC.

### Src phosphorylates and alters KRAS switch conformation

**Fig. 1:**
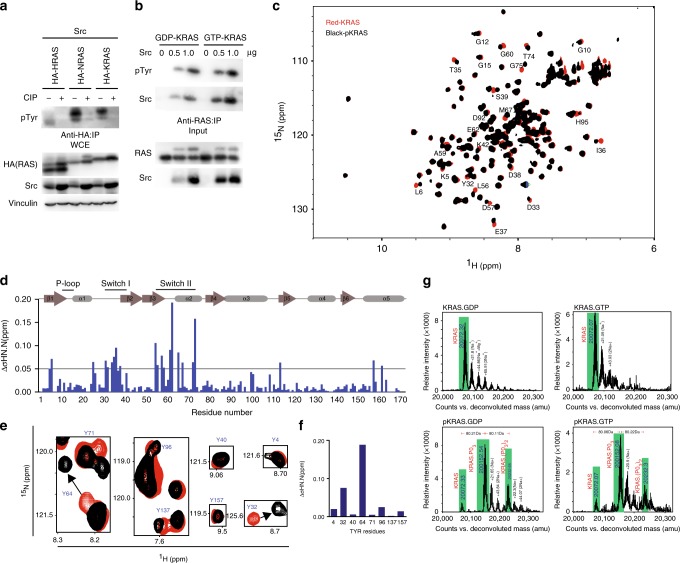
Src phosphorylation of KRAS alters the structure of the switch regions. **a** HEK293 cells transfected with the indicated plasmids were lysed, treated with (+) or without (−) calf intestinal alkaline phosphatase (CIP), immunoprecipitated, and immunoblotted with the indicated antibodies. **b** Purified recombinant human GDP- and GTP-bound KRAS protein were subjected to in vitro kinase assay using purified recombinant Src^cat^ kinase and were immunoprecipitated and immunoblotted with the indicated antibodies. **c** An overlay of 2D ^1^H-^15^N HSQC spectra of unmodified KRAS-GDP (red) and pKRAS-GDP phosphorylated by Src (black). The labeled residues are residues with notable chemical shift perturbation. **d** Histogram mapping the backbone NH chemical shift changes caused by Src phosphorylation. Chemical shift changes of the N and NH peaks of the unmodified vs. phosphorylated cross-peaks in the 2D ^1^H-^15^N HSQC spectra were calculated using the formula ∆δ NH.N(ppm) = √(〖 (∆H)〗^2 + 〖(∆N/5)〗^2) and plotted against residue number. **e** Overlay of ^1^H-^15^N HSQC spectra of ^15^N-KRAS tyrosine peaks before (red) and after (black) phosphorylation. The residue numbers are indicated in blue and the arrows indicate the direction of the chemical shift change. Full spectra are shown in **c**. **f** Chemical shift changes of the KRAS tyrosine residues induced by phosphorylation. **g** Upper panel: Mass spectra of unmodified GDP-loaded KRAS (left) and KRAS loaded with the non-hydrolyzable GTP analog GMPPNP (right). The major peak of KRAS is shaded green, and the higher molecular weight peaks (+22 Da) correspond to sodium adducts (the predicted molecular mass of ^15^N-KRAS (residues 1–173 C118S) is 20074.35 Da). Lower panel: Mass spectrum of GDP-loaded pKRAS (left) and GTP-loaded pKRAS (right) following treatment with Src in vitro. The three peaks highlighted in green correspond to unmodified KRAS, KRAS phosphorylated at one site (+80 Da) and KRAS phosphorylated at two sites (+160 Da). The immunoblot data are representative of at least three independent experiments

Phosphorylation of a protein alters the chemical environment and thus the chemical shifts of the modified residue, as well as spatially or sequentially adjacent residues. Ultimately, phosphorylation can induce conformational changes that affect protein function^16^. We first asked whether KRAS is phosphorylated by Src. In human embryonic kidney epithelial HEK293 cells transfected with plasmids encoding Src in combination with HA-tagged HRAS, NRAS, or KRAS WT, pKRAS was detected at levels similar to pHRAS and pNRAS, which was abolished when lysates were treated with calf intestinal alkaline phosphatase (Fig. 1a). In contrast, another small GTPase protein, RAC2, exhibited negligible tyrosyl phosphorylation in the presence of Src (Supplementary Fig. 4a). Other tyrosine kinases, focal adhesion kinase (FAK) and SYK failed to phosphorylate KRAS while WT, but not catalytically impaired, Src effectively phosphorylated KRAS (Supplementary Fig. 4b). The ubiquitin ligase CBL, which has been shown to target Src for ubiquitin-mediated destruction^17^, had negligible effect on KRAS phosphorylation (Supplementary Fig. 4b). Furthermore, purified KRAS loaded with either GDP or GTP was phosphorylated to a similar extent by recombinant Src catalytic domain (Src^cat^) in vitro (Fig. 1b). These results suggest that KRAS is phosphorylated specifically via Src, which can be reversed by a phosphatase.

We next used NMR to examine potential conformational changes in KRAS induced by phosphorylation by examining chemical shift perturbations in the ^1^H-^15^N heteronuclear single quantum correlation (HSQC) spectra of isotopically labeled ^15^N KRAS-GDP in the presence of excess ATP upon addition of a small amount of catalytically active Src^cat^ (Src to KRAS molar ratio of 1:250) (Fig. 1c, d). Phosphorylation induced chemical shift perturbations on numerous KRAS residues, particularly those in the two switch regions, of magnitude consistent with Tyr phosphorylation^16^. The most perturbed residues were Tyr32, Asp33, Thr35, Ile36, and Glu37 in switch I and Leu56, Asp57, Ala59, Gly60, Tyr64, Met67, and Gly75 in switch II. Notably, Tyr32 and Tyr64 exhibited much larger chemical shift perturbations than the other six tyrosine residues in KRAS (Fig. 1e, f). The mass spectrum of intact KRAS following Src^cat^ phosphorylation exhibited a major peak corresponding to singly phosphorylated KRAS (+80 Da), a smaller peak for doubly phosphorylated KRAS (+160 Da) and a minor peak corresponding to unmodified KRAS, independent of the nucleotide bound (Fig. 1g). Other minor peaks in the mass spectra were assigned to sodium adducts.

### KRAS is phosphorylated via Src on conserved Tyr32 and Tyr64

**Fig. 2:**
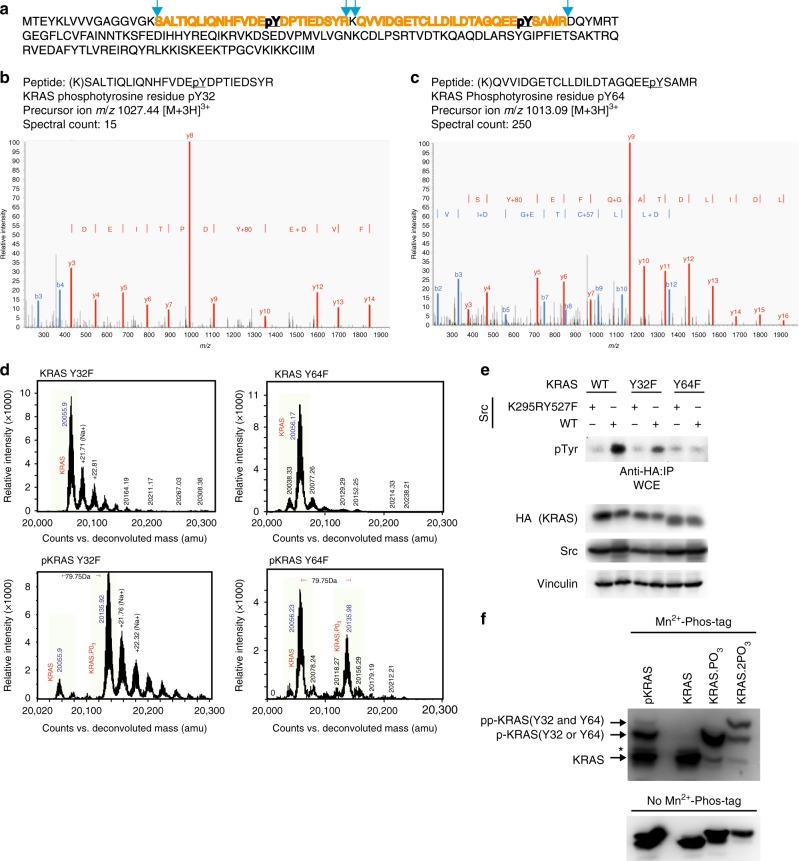
Src phosphorylates KRAS tyrosine 32 and 64. **a**–**c** Two phosphorylated tyrosine residues from the KRAS protein sequence (**a**) were identified from in vitro kinase reaction samples that were reduced/alkylated, trypsin-digested, and analyzed by LC-MS. MS/MS spectra matched indicated KRAS tryptic peptides containing residue Y32 (**b**) and Y64 (**c**). **d** Upper panel: Mass spectra of unmodified KRAS^Y32F^ (left) and KRAS^Y64F^ (right). Lower panel: Mass spectrum of pKRAS^Y32F^ (left) and pKRAS^Y64F^ (right). The major peak of KRAS is shaded light green. **e** HEK293 cells were transfected with the indicated plasmids. Cells were lysed, immunoprecipitated, and immunoblotted with the indicated antibodies. The immunoblot data are representative of at least three separate experiments. **f** GTP-bound KRAS were subjected to in vitro kinase assay using purified Src kinase (left lane) and were separated into un-phosphorylated (KRAS), mono-phosphorylated (KRAS.PO_3_), and di-phosphorylated (KRAS.2PO_3_) forms by anion exchange chromatography. These samples were then analyzed by 50 μM Mn^2+^-PhosTag SDS-PAGE (upper panel) or conventional SDS-PAGE (lower panel) and immunoblotted with anti-RAS antibody. Asterisk indicates non-specific band. IP immunoprecipitation, WCE whole-cell extract. The immunoblot data are representative of at least three independent experiments

To unambiguously identify the site(s) of phosphorylation, we conducted liquid chromatography (LC)-MS/MS sequencing of trypsin-digested KRAS following phosphorylation by Src. The LC-MS/MS data identified a tryptic peptide containing pTyr64 as the most abundant tyrosyl phosphorylated species, followed by one containing pTyr32, whereas the abundance of other phosphorylated peptides was negligible (Fig. 2a–c). Mass spectra of intact KRAS^Y32F^ and KRAS^Y64F^ mutants following Src^cat^ phosphorylation each lacked doubly phosphorylated KRAS, while Y32F exhibited a major peak corresponding to singly phosphorylated KRAS and Y64F produced two peaks of similar intensity corresponding to un-phosphorylated and singly phosphorylated species (Fig. 2d). Consistent with these results, the cellular levels of tyrosyl phosphorylation of KRAS co-expressed in HEK293 cells with Src were reduced for Y32F and dramatically lower for the Y64F mutant in comparison to WT KRAS (Fig. 2e). We further separated and enriched the mono-phosphorylated and di-phosphorylated forms of KRAS via anion exchange chromatography (Supplementary Fig. 5) and observed using Mn^2+^-PhosTag sodium dodecyl sulfate-polyacrylamide gel electrophoresis (SDS-PAGE)^18^, two distinct slower migrating bands (i.e., mono- and di-phosphorylated), indicating that the modifications are tyrosyl phosphorylations catalyzed by Src (Fig. 2f). These results unambiguously identify Tyr32 and Tyr64 within switch I and II, respectively, as major phosphorylation acceptor sites on KRAS.

### SHP2 dephosphorylates KRAS and promotes downstream signaling

**Fig. 3:**
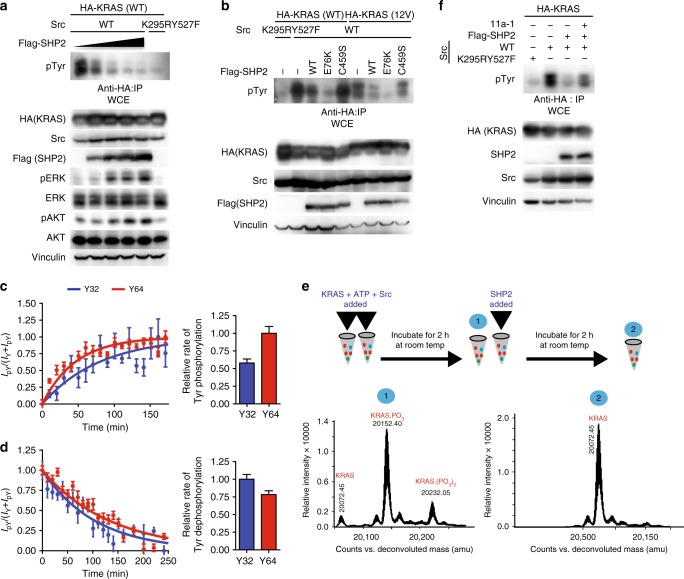
SHP2 dephosphorylates tyrosyl phosphorylated KRAS. **a**, **b** HEK293 cells were transfected with the indicated plasmids. Cells were lysed, immunoprecipitated, and immunoblotted with the indicated antibodies. **c** Phosphorylation was initiated by addition of 2 μM of Src to 250 μM of KRAS in the presence of 2 mM ATP and 1 mM activated sodium vanadate. Sequential ^1^H-^15^N HSQC NMR spectra were collected, and peaks from un-phosphorylated and phosphorylated Tyr32 (blue) and Tyr64 (red) were integrated from each spectrum to plot curves of the fraction phosphorylated vs. time for each site. The error bars correspond to the noise-to-signal ratio of the respective peaks. The relative rates of phosphorylation of each site determined from a representative experiment (performed in duplicate) by curve fitting are shown in histograms on the right. The error bars indicate the standard curve fitting error obtained from the fitting analysis. **d** SHP2 (2 μM) was added to Src-phosphorylated KRAS sample purified in the presence of vanadate, and sequential ^1^H-^15^N NMR spectra were collected to monitor dephosphorylation. Similar to **c**, the fractions of phosphorylated Tyr32 (blue) and Tyr64 (red) were plotted and rates of dephosphorylation were determined and are shown in the histograms. Rate curves from a representative experiment (performed in duplicate) were fitted to one phase exponential decay/association functions using the GraphPad Prism 4.0 software. Histogram error bars indicate the standard curve fitting error obtained from the fitting analysis. **e** Top panel; experimental set-up whereby two tubes containing 200 μM of KRAS and 3 μM of Src were incubated in in the presence of 2 mM ATP for 2 h (in the absence of vanadate). Sample 1 was then snap frozen and stored at −80 °C for MS analysis, and 3 μM of SHP2 was added to the remaining tube and incubated for an additional 2 h. Sample 2 was then frozen. Bottom panels; MS spectra of samples 1 and 2 with phosphorylation state of each mass indicated. **f** HEK293 cells transfected with the indicated plasmids were treated with (+) or without (−) 10 μM of 11a-1, lysed, immunoprecipitated, and immunoblotted with the indicated antibodies. The immunoblot data are representative of at least three independent experiments

We showed previously that SHP2 dephosphorylates pH/NRAS^6^ and that alkaline phosphatase dephosphorylates Src-induced tyrosyl pKRAS (Fig. 1a). Thus we asked whether SHP2 dephosphorylates pKRAS. Ectopic Flag-SHP2 expression was associated with decreased levels of Src-induced HA-pKRAS, together with increased levels of downstream pERK and pAKT signals in a dose-dependent manner (Fig. 3a). The constitutively active disease-causing Flag-SHP2^E76K^ mutant markedly reduced the level of pKRAS WT and G12V, while the catalytically dead SHP2^C459S^ mutant had a negligible effect (Fig. 3b). Further, we investigated the dephosphorylation of pKRAS in vitro using NMR and MS. Both approaches demonstrated that SHP2 dephosphorylates Src-phosphorylated KRAS (Fig. 3c–e). Notably, inhibition of SHP2 using 11a-1 attenuated SHP2-mediated dephosphorylation of pKRAS (Fig. 3f).

### Endogenous RAS phosphorylation is regulated by Src and SHP2

**Fig. 4:**
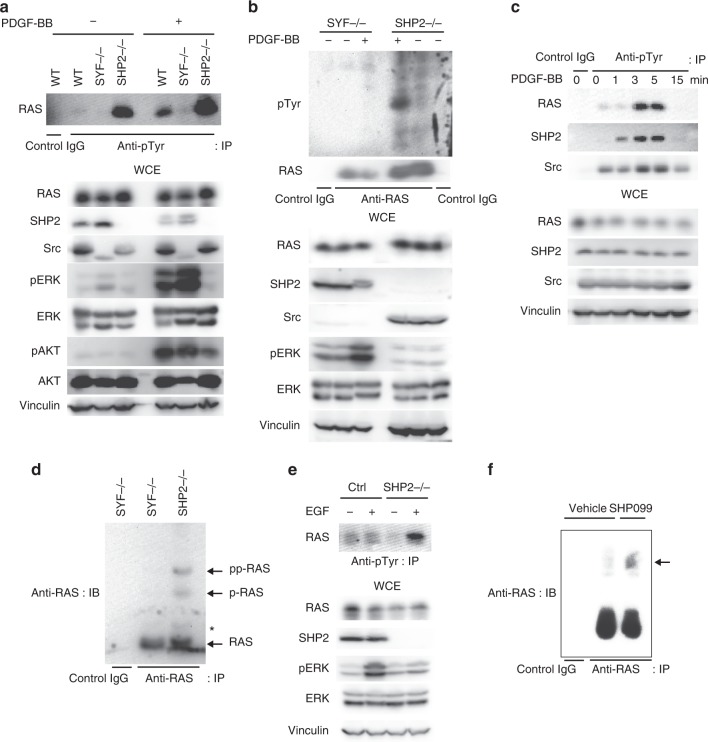
Endogenous RAS phosphorylation is regulated temporally by Src and SHP2. **a** WT, *Src/Yes/Fyn (SYF)−/−* and CRISPR/Cas9-mediated *SHP2−/−* MEFs were serum starved and treated with 20 ng/ml of PDGF-BB. Equal amounts of lysates were immunoprecipitated with isotype-matched antibody or anti-pTyr antibody and immunoblotted with the indicated antibodies. **b** *SYF−/−* and *SHP2−/−* MEFs were serum starved and treated with 20 ng/ml of PDGF-BB. Equal amounts of lysates were immunoprecipitated with isotype matched antibody or anti-RAS antibody and immunoblotted with the indicated antibodies. **c** MEFs were serum starved and treated with 20 ng/ml of PDGF-BB or were left untreated for the indicated periods of time. Equal amounts of lysates were immunoprecipitated with isotype-matched antibody or anti-pTyr antibody and immunoblotted with the indicated antibodies. **d** *SYF−/−* and *SHP2−/−* MEF lysates were immunoprecipitated with isotype-matched antibody or anti-RAS antibody and analyzed by 50 μM Mn^2+^-PhosTag SDS-PAGE. Asterisk indicates non-specific band. **e** CRISPR/Cas9-mediated non-target (Ctrl) or *SHP2−/−* CFPAC1 cells were serum starved and treated with (+) or without (−) 10 ng/ml of EGF. Equal amounts of lysates were immunoprecipitated and immunoblotted with the indicated antibodies. **f** OCIP.343 xenograft tumor lysates from the indicated treatment groups were immunoprecipitated with isotype-matched antibody or anti-RAS antibody and analyzed by Mn^2+^-PhosTag SDS-PAGE. Arrow indicates a slower migrating band. The immunoblot data are representative of at least three independent experiments

We observed tyrosyl phosphorylation of endogenous RAS in WT mouse embryonic fibroblasts (MEFs) following platelet-derived growth factor (PDGF) stimulation (Fig. 4a). Furthermore, *Src/Fyn/Yes* knockout (*SYF−/−*) MEFs showed negligible levels of endogenous pRAS while *SHP2−/−* MEFs displayed markedly enhanced levels of endogenous pRAS, which was further increased in the presence of PDGF treatment (Fig. 4a). The amount of RAS phosphorylation was inversely associated with downstream pERK and pAKT levels (Fig. 4a), supporting the notion that phosphorylation of RAS impairs downstream signaling. Similar results were observed in a reciprocal experiment in which the noted lysates were immunoprecipitated with anti-RAS antibody followed by anti-pTyr immunoblot (Fig. 4b). Importantly, inhibition of Src using the non-ATP competitive substrate pocket-directed Src inhibitor KX2-391^19^ or Src-specific small interfering RNA (siRNA) led to increased levels of pERK in *SHP2−/−* MEFs (Supplementary Fig. 6a, b), suggesting that the observation of attenuated downstream of RAS signaling in *SHP2−/−* MEFs is dependent, at least in part, on Src activity. The amplitude and duration of endogenous RAS phosphorylation as well as SHP2 and Src phosphorylation following growth factor stimulation were strongly and rapidly enhanced within 5 min (Fig. 4c). Notably, two slower migrating bands of endogenous RAS (consistent with di- and mono-phosphorylated forms as shown in Fig. 2f using purified KRAS) were detected by Mn^2+^-PhosTag SDS-PAGE in *SHP2−/−* MEFs, which were absent in *SYF−/−* MEFs (Fig. 4d). Similarly, *SHP2−/−* CFPAC1 harboring *KRAS*^*G12V*^ mutation showed markedly enhanced levels of tyrosyl-phosphorylated endogenous RAS in response to EGF (Fig. 4e). Lysates of PDAC tumors treated with SHP099 or vehicle only (Supplementary Fig. 3) were analyzed by Mn^2+^-PhosTag SDS-PAGE. The slower migrating RAS band was stronger in the SHP099 treatment group (Fig. 4f), suggesting that the inhibition of SHP2 via SHP099 curtailed dephosphorylation of pRAS in vivo. These results demonstrate that endogenous RAS is singly or doubly tyrosyl phosphorylated and that the phosphorylation-mediated RAS GTPase cycle is a tightly controlled dynamic process.

### Tyrosyl phosphorylation of KRAS stalls its GTPase cycle

**Fig. 5:**
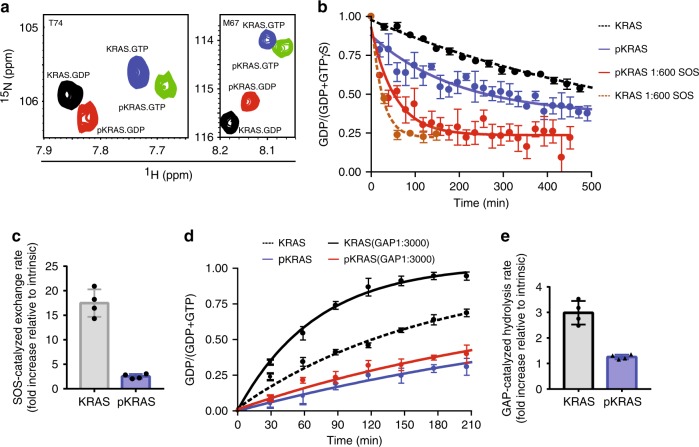
Tyrosyl phosphorylation of KRAS disrupts GTPase cycle. **a** Overlay of four ^1^H-^15^N HSQC spectra of two representative residues T74 and M67, in which the four peaks represent GDP-loaded KRAS (black), GTP-loaded KRAS (blue), GDP-loaded Src-phosphorylated (p)KRAS (red), and GTP-loaded pKRAS (green). **b** Real-time NMR-derived nucleotide exchange curves for unmodified vs. Src-phosphorylated KRAS. A 250μM sample of GDP-loaded ^15^N KRAS (black) or pKRAS (blue) was incubated with ten-fold molar excess of GTPγS. Each dot represents mean fraction of KRAS that is loaded with GDP on the basis of peak intensities [*I*_GDP_/(*I*_GDP_ + *I*_GTPɣS_)] from the same three residues of KRAS and pKRAS. SOS^cat^ was added at a ratio of 1:600 to KRAS (orange) or pKRAS (red). **c** Src phosphorylation of KRAS impairs SOS^cat^-assisted nucleotide exchange. Fold increase in the rate of exchange upon addition of SOS^cat^ to KRAS or pKRAS. The reaction was performed twice in the absence and twice in the presence of SOS^cat^ and four ratios were determined from each pairwise comparison. **d** GTP hydrolysis curves illustrating intrinsic and RASA1 GAP domain (1:3000 ratio) assisted GTP hydrolysis for unmodified KRAS and pKRAS. **e** Src phosphorylation of KRAS reduces sensitivity to GAP activity. Fold increase in the rate of GTP hydrolysis with addition of RASA1 GAP domain for KRAS vs. pKRAS. The reaction was performed twice in the absence and twice in the presence of RASA1 GAP domain and four ratios were determined from each pairwise comparison. **b**, **d** present a single representative experiment that was repeated at least twice. Error bars represent the standard deviation of the fraction GDP as reported by three pairs of cross-peaks. Error bars in **c**, **e** indicate standard deviation of the fold changes

To interrogate the impact of tyrosyl phosphorylation on the KRAS GTPase cycle, we used a real-time NMR GTPase assay^20,21^ to measure nucleotide exchange and hydrolysis rates of unmodified KRAS and pKRAS. For the exchange assay, GDP-loaded ^15^N KRAS or pKRAS were mixed with a ten-fold molar excess of GTPγS in an NMR tube, and consecutive ^1^H-^15^N HSQC spectra were acquired as the exchange reaction proceeded. The intensities of peaks from residues whose chemical shifts are sensitive to GDP/GTP binding were used to calculate the rate of the nucleotide exchange (Fig. 5a). Interestingly, phosphorylation of KRAS increased the intrinsic rate of nucleotide exchange by ~2.6-fold, while reducing the sensitivity of pKRAS to the exchange activity of the catalytic domain of the GEF SOS1 (SOS^cat^)^22^ (Fig. 5b, c and Supplementary Table 1a). Specifically, the addition of recombinant SOS^cat^ to unmodified KRAS at a ratio of 1:600 accelerated the exchange reaction 17-fold, whereas nucleotide exchange of pKRAS was enhanced by <3-fold (Fig. 5b, c and Supplementary Table 1a). The activation of KRAS^G12V^ mutant by SOS was similarly impaired upon phosphorylation (Supplementary Fig. 7a). Thus, while phosphorylation of KRAS by Src mildly increases the intrinsic nucleotide exchange rate, these posttranslational modifications impair its activation by SOS by almost an order of magnitude.

We next loaded KRAS or pKRAS with GTP and monitored its hydrolysis by real-time NMR. pKRAS exhibited an intrinsic GTP hydrolysis rate that was ~3-fold lower than that of unmodified KRAS (Fig. 5d, e and Supplementary Table 1b). In the presence of 1:3000 molar ratio of recombinant GAP domain of RASA1, the rate of GTP hydrolysis of unmodified KRAS was accelerated by >300% while that of pKRAS increased by <20% (Fig. 5d, e and Supplementary Table 1b). KRAS^G12V^ mutant has, however, impaired intrinsic hydrolysis and is insensitive to GAP stimulation, thus phosphorylation had little impact on these rates (Supplementary Fig. 7b). These results indicate that phosphorylation of KRAS alters its intrinsic nucleotide exchange and hydrolysis rates in a manner that favors accumulation of pKRAS-GTP, while simultaneously rendering it resistant to inactivation by GAP activity and largely insensitive to GEF activity, thus uncoupling pKRAS from upstream regulation.

### Tyrosyl phosphorylation of KRAS impairs effector RAF binding

**Fig. 6:**
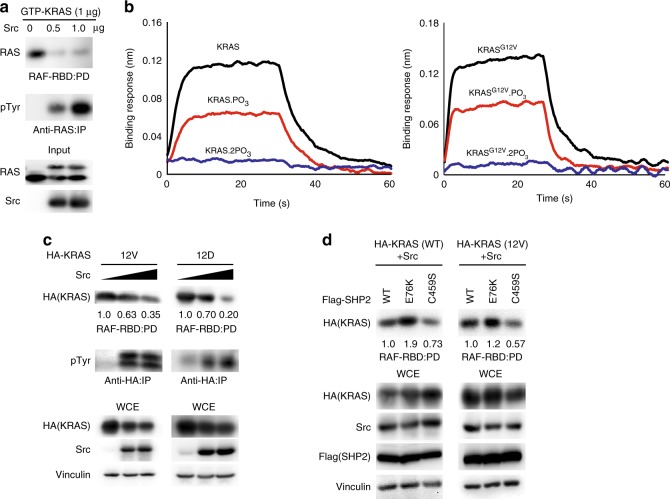
Tyrosyl phosphorylation of KRAS attenuates binding to RAF. **a** Purified recombinant human GTP-bound KRAS protein was subjected to in vitro kinase assay using purified recombinant Src kinase and pulled down using Raf-1:RBD-conjugated beads or immunoprecipitated and immunoblotted with the indicated antibodies. **b** The binding of un-, mono-, and di-phosphorylated KRAS, each at a concentration of 250 nM, to immobilized GST-BRAF-RBD was measured using biolayer interferometry (Octet). The full binding curves at a range of KRAS concentrations is shown in Supplementary Fig. 8. Left panel: wild-type KRAS. Right panel: KRAS^G12V^ mutant. **c**, **d** HEK293 cells were transfected with the indicated plasmids. Cells were lysed, pulled down using Raf-1:RBD-conjugated beads, or were immunoprecipitated and immunoblotted with the indicated antibodies. Numerical values indicate the ratio of quantified signals over the control (lane 1 of each panel; set at 1.0) determined by densitometry using the ImageJ software. The immunoblot data are representative of at least three independent experiments. PD pulldown

We asked whether phosphorylation of KRAS via Src influenced its binding to effector proteins. Tyrosyl phosphorylation of purified GTP-loaded KRAS via Src markedly attenuated the interaction with RAF-RBD in pull-down assays (Fig. 6a). To quantify the effect of phosphorylation on this interaction, we performed biolayer interferometry using un-phosphorylated, mono-phosphorylated, and di-phosphorylated KRAS, which were separated and enriched via anion exchange chromatography (Supplementary Fig. 5). Un-phosphorylated KRAS bound with a *K*_D_ value of ~ 300 nM, whereas the mono-phosphorylated fraction, which contains a mixture of pTyr32 and pTyr64 KRAS, exhibited ~2-fold weaker binding, and di-phosphorylated KRAS exhibited severely impaired binding to BRAF-RBD and much higher concentrations were required to detect its binding (Fig. 6b and Supplementary Fig. 8a–c). The high protein concentrations promoted some non-specific interactions that reduced the accuracy of *K*_D_ determination; however, di-phosphorylation of KRAS reduced its affinity for BRAF by at least 15-fold. Notably, the oncogenic KRAS^G12V^ and KRAS^G12D^ mutants were also phosphorylated by Src in a dose-dependent manner, which was associated with markedly attenuated RAF-RBD binding (Fig. 6b, c). Moreover, the dephosphorylation status of KRAS WT or 12V was associated with enhanced binding to RAS-binding domain (RBD) of RAF (Fig. 6d). These results demonstrate that phosphorylation of WT or oncogenic KRAS mutants attenuates its binding to effector RAF.

Moreover, our finding that pKRAS has impaired affinity for RAF-RBD has an important implication for the most widely used assay of RAS activation (i.e., the RBD pull-down assay). It has been consistently reported that inhibition of SHP2 reduces the amount of RAS-GTP pulled down in such assay, which has been interpreted as reduced RAS activation due to impaired SOS activation or increased GAP activity. However, our results demonstrate that phosphorylation of RAS-GTP upon SHP2 inhibition can also contribute to reducing RAS binding to RBD.

## Discussion

SHP2 is a well-established major regulator of RAS-to-RAF-to-MAPK signal pathway and somatic gain-of-function *SHP2* mutations or overexpression have also been identified in several hematologic malignancies as well as solid tumors such as gastric, breast, lung and pancreatic cancers and RASopathies^1,2,6,23–25^. These observations have led to the notion that SHP2 could be used as prognostic marker and potentially targeted for therapy even prior to our reporting of its direct involvement in the regulation of RAS^6^. Although we and others have shown that the treatment of RAS-dependent cancers can benefit from SHP2 inhibition^6–11^, the molecular mechanisms that relate SHP2 to RAS signaling have remained unclear and controversial^10^.

SHP2 is a phosphatase containing two SH2 domains, a catalytic PTP domain and a C-terminal regulatory tail with sites of tyrosyl phosphorylation. At basal state, SHP2 adopts a closed inactive conformation in which its SH2 and PTP domains are occluded^26^. In the activated open state, the PTP domain of SHP2 is exposed to substrates and the SH2 domains are available to interact with pTyr residues. Phosphorylated SHP2 can recruit GRB2 and SOS to phosphorylated receptor tyrosine kinases, and the docking of SHP2 SH2 domains to substrates may promote the catalytic function of the PTP domain^27,28^. Several lines of evidence suggest the importance of the catalytic function of SHP2 in regard to its role for RAS activation, although the relevant targets are still debated^29^. SHP2 has been implicated as a regulator of RAS GTPase cycle by regulating GEF and GAP localization by dephosphorylating the p120 RASGAP docking sites on EGFR and GAB1^30,31^ or GRB2-binding sites on Sprouty, reversing its negative regulation of GRB2/SOS recruitment^32^. These models, which are not necessarily mutually exclusive with the presented model, involve SHP2 regulation of the RAS GTPase cycle by modulation of the regulators of RAS.

**Fig. 7:**
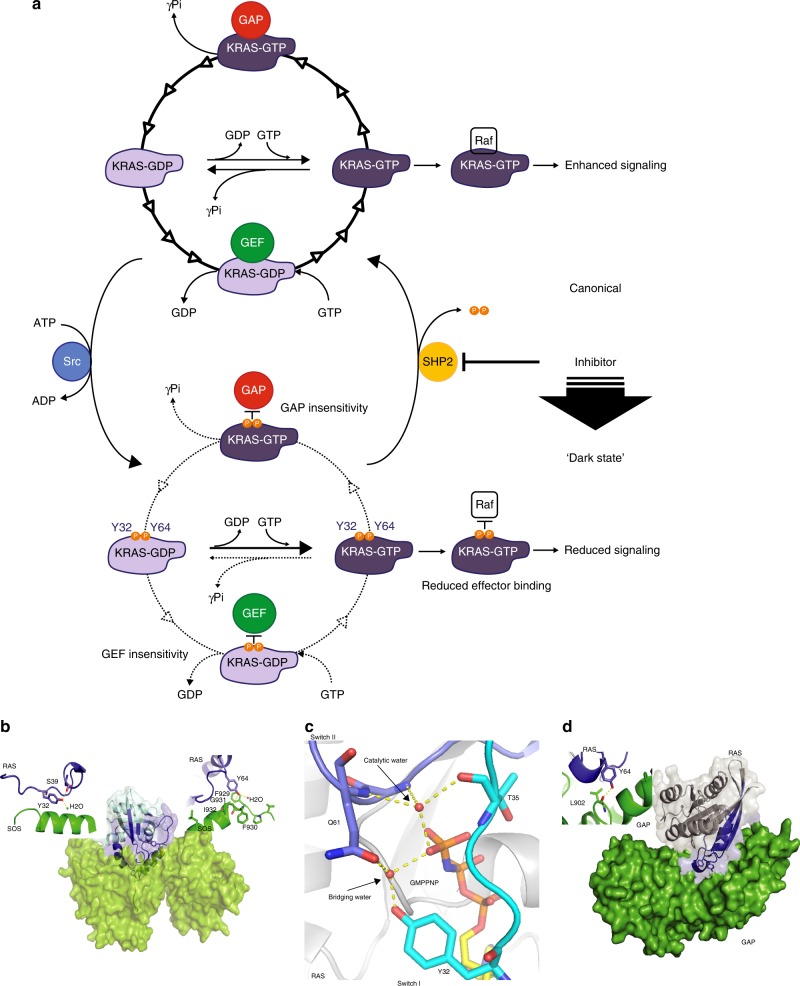
Model of the impact of phosphorylation on KRAS GTPase cycle and structural rationale. **a** Phosphorylation decouples KRAS from effectors and GTPase cycle regulation. The GTPase cycle of unmodified KRAS is shown on top. Nucleotide exchange and hydrolysis are accelerated by the activities of GEFs and GAPs, respectively. GTP-loaded KRAS binds and activates RAF. Src phosphorylation of Tyr32 and Tyr64 of KRAS-GDP or KRAS-GTP alters all steps in this GTPase cycle (lower part). Upon phosphorylation, KRAS becomes insensitive to regulation by both GEFs and GAPs, whereas intrinsic nucleotide exchange is enhanced and intrinsic GTP hydrolysis is impaired. This leads to the accumulation of phosphorylated KRAS-GTP, which has reduced affinity for RAF, thus phosphorylation limits KRAS signaling. SHP2 dephosphorylates KRAS and thereby unleashes KRAS-GTP to rapidly activate RAF. Inhibition of SHP2 promotes accumulation of phosphorylated KRAS, dampening RAF signaling and subsequently suppressing oncogenesis. Line thickness and number of arrowheads represent relative reaction rates of each step. **b** Ribbon diagram SOS (green) contact with Tyr32 and Tyr64 of RAS (PDB ID 1BKD). **c** Proposed coordination of switch I and switch II RAS residues in intrinsic GTP hydrolysis (PDB ID 4G0N). **d** Switch I and II (blue) of RAS are the main interaction site with GAP (green) (PDB ID 1WQ1)

Although the biological significance remains unclear, phosphorylation of the residues homologous to Tyr32 on RHOA (Tyr34), RAB24 (Tyr37), and RAN (Tyr39) has been described^33–35^ and Src has also been shown to phosphorylate CDC42, R-RAS, and the heterotrimeric G protein α subunit^36–38^. There are also examples of phosphorylation affecting the molecular switch function of RAS. For example, HRAS Tyr137 phosphorylation by Abelson tyrosine protein kinase has been shown to allosterically enhance the binding of HRAS to effector RAF1^39^ while phosphorylation of a plant GTPase Toc34 is thought to affect its GTPase activity^40,41^. Here we show that endogenous RAS is tyrosyl phosphorylated and that KRAS is phosphorylated by Src on Tyr32 and Tyr64 in a kinase- and site-specific manner. We show that tyrosyl phosphorylation of KRAS alters the conformation of switch I and II, consequently attenuating GEF-mediated nucleotide exchange, GAP-assisted GTP hydrolysis, and the binding affinity of KRAS to the effector RAF (Fig. 7a). These results demonstrate that phosphorylation of KRAS stalls the GTPase cycle into a “dark state” while SHP2-mediated dephosphorylation is required to maintain dynamic cycling or to unleash signaling-competent KRAS from the dark state of pKRAS. Importantly, KRAS mutations frequently occur at codon 12 and substitution mutations encoding G12D and G12V are among the most prevalent in PDAC tumors^2,42^. Notably, we show that Src- and SHP2-mediated regulation of KRAS extends to these common KRAS mutants and that the inhibition of SHP2 activity disrupts the phosphorylation cycle, shifting the equilibrium of oncogenic KRAS toward the dark state (Fig. 7a). Although it is formally unknown the stoichiometry of KRAS phosphorylation in cells, it appears reasonable that dynamic (de)phosphorylation of KRAS in the appropriate spatiotemporal context upon external stimulus would be sufficient to trigger downstream signaling. Src and SHP2 both have well-established functions at receptor tyrosine kinases and are thus poised to regulate the phosphorylation of the local KRAS that is most relevant to signaling (i.e., KRAS with the potential to become activated by SOS at activated receptors).

A crystal structure of RAS in complex with SOS reveals that both Tyr32 and Tyr64 form points of contact with SOS. The mechanism underlying the GEF activity of SOS involves the insertion of a helix from the Cdc25 domain of SOS into the RAS nucleotide-binding domain to open up the two switch regions to destabilize nucleotide binding. This helical interface includes a direct interaction between Tyr32 and Asn944 of SOS (PDB: 1BKD), and the Tyr32 hydroxyl forms a hydrogen bond with Ser39 in switch I and a water molecule, which appears to stabilize the open conformation of RAS^43^ (Fig. 7b). Meanwhile, Tyr64 inserts into a hydrophobic pocket on SOS where its hydroxyl forms a hydrogen bond with the backbone of Gly931 (Fig. 7b). Mutations of either Tyr32 or Tyr64 have been shown to disrupt interaction with SOS and impair the sensitivity of RAS to its catalytic activity^44,45^, suggesting that the reduced sensitivity of pKRAS to SOS is likely due to disruption of interactions with either Tyr hydroxyl in combination with steric hindrance and unfavorable electrostatics of the phosphate group.

Intrinsic GTP hydrolysis by RAS involves attack on the γ-phosphate by a catalytic water molecule^46,47^, and RAS mutations that perturb the activation of the catalytic water molecule reduce the intrinsic hydrolysis rate^48–50^. This water molecule interacts with the γ-phosphate of GTP, which is stabilized by a second “bridging” water molecule that interacts directly with the side-chain hydroxyl of Tyr32 (Fig. 7c), suggesting that this coordination would likely be spoiled by the addition of a phosphate moiety to the Tyr32 hydroxyl. As the same water molecule participates in GAP-catalyzed GTP hydrolysis, the effect of phosphorylation on its coordination may partially explain the reduced sensitivity to GAP activity. Further, the crystal structure of RAS in complex with the GAP domain of RASA1 revealed extensive interaction with both switch I and II, and in particular Tyr64, which forms a hydrophobic interface between RAS and the GAP domain^51^ (Fig. 7d). Moreover, anionic substitutions of Tyr64 (Y64D/E) disrupt GAP-assisted hydrolysis^52,53^, supporting the notion that Tyr64 phosphorylation would impair interaction with the GAP.

Another important aspect of KRAS phosphorylation is that tyrosyl phosphorylation of KRAS at Tyr32 and Tyr64 reduces the affinity to RAF, which renders direct shut down of KRAS-mediated MAPK pathway. The crystal structure of RAS in complex with RAF demonstrates that switch I forms the core of the binding site^54^. Notably, Tyr32 is found in the middle of the RAS effector-binding site, suggesting how phosphorylation of this residue reduces the affinity to RAF, thereby attenuating the downstream RAF-to-MEK-to-ERK pathway. However, considering that both switch I and II regions are critical for binding multiple effector proteins, discerning the relative contribution of Tyr32 and/or Tyr64 modification on individual effector recruitment will be important for understanding how RAS phosphorylation governs the signaling of a myriad of pathways.

Here we show that Src phosphorylation decouples KRAS from regulation by GEFs and GAPs, leading to the accumulation of a GTP-bound “dark state” that is impaired in its ability to engage and activate RAF kinase. While a large body of work has established pro-oncogenic roles for Src in tumor development and metastasis, its phosphorylation of RAS is distinctively a tumor-suppressive function. Although preclinical studies implicated Src as a therapeutic target for cancer, phase II trials failed to demonstrate a significant clinical benefit of Src inhibitor monotherapy for metastatic solid tumors including breast, prostate, head and neck, colon, pancreatic, or non-small cell lung cancers^55^. These clinical trials, however, were performed in unselected patients, lacking effective response biomarkers to guide the design of clinical trials, which was partly due to the enormous complexity of Src signaling. Recent preclinical data suggest that cells with elevated Src activity are more likely to respond to Src inhibition, while tumors with diminished Src signaling resulting from alternative oncogenic pathways may contribute to de novo resistance to Src inhibitors^56–59^. These, albeit limited, studies demonstrate that hyperactive Src signaling may potentially serve as a biomarker for successful targeting of Src and clinical efficacy. However, other molecular alterations in cancers may impact on the response of cancer cells to Src inhibitors. For example, c-MET amplification in gastric cancer as well as the autophagy pathway were shown to promote resistance to Src inhibitors^59,60^ while acquired resistance of ER+ breast cancers to the Src family kinase inhibitor saracatinib is associated with the reactivation of the mammalian target of rapamycin pathway^61,62^. These results suggest that cancer cells can acquire resistance to Src inhibitor via multiple genetic alterations. Thus further clinical studies are needed to develop more reliable biomarkers that can guide clinical trials. To our knowledge, KRAS mutational status has not been evaluated as a biomarker for response to Src inhibitors. In light of our present study suggesting tumor-suppressive role of Src in the context of oncogenic KRAS as well as recent reports demonstrating clinical utility of SHP2 inhibitors in the management of mutant KRAS-driven cancers^7–11^, inclusion of KRAS mutational status would be prudent for future clinical trials of Src and SHP2 inhibitors as mono or combination therapies.

## Methods

### Cells

HEK293, MEF, MEF-SYF(−/−), CFPAC1, Capan-1, HPAF-II, SW1990, HUPT3, and MiaPaCa-2 cells were obtained from the American Type Culture Collection. P411T1 and PancT6 were generated from PDAC PDXs^42^. HEK293, MEF, MEF-SYF(−/−), and MiaPaCa-2 cells were maintained in Dulbecco’s modified Eagle’s medium (DMEM; Invitrogen) supplemented with 10% (v/v) heat-inactivated fetal bovine serum (FBS; Wisent) at 37 °C in a humidified 5% CO_2_ atmosphere. CFPAC1, Capan-1, HPAF-II, SW1990, HPAC, and HUPT3 cells were maintained similarly in RPMI-1640 (Wisent) medium supplemented with 10% (v/v) FBS. P411T1 and PancT6 cells were maintained in DMEM/F12 medium (Thermo Fisher Scientific, 11330–032) supplemented with 5 ng/ml EGF (R&D Systems, 236-EG-01M), 10 μg/ml insulin (Thermo Fisher Scientific, 12585–014), and 10% (v/v) FBS.

### Plasmids

A plasmid encoding human pBabe-KRAS4B WT was generously provided by Channing Der (University of North Carolina, Chapel Hill), which was subcloned into pcDNA3 using KpnI and NotI to integrate an N-terminal HA tag. KRAS mutants (G12V, Y32F and Y64F) were generated by site-directed mutagenesis. Human pcDNA3-HA-HRAS, pCGN-HA-NRAS, pcDNA3-Flag-RAC2, pCDNA-HA-FAK P712/715A, pCDNA-HA-FAK Y576/577F, pMSCV-mCherry-SYK, pCMV5-Src (WT or K295RY527F), and pCMV5-SHP2 (WT, E76K or C459S) were obtained from Addgene. Flag-SHP2 constructs were subcloned into pcDNA3 and a plasmid encoding HA-CBL was subcloned into the pcDNA-DEST4.0 vector using Gateway Cloning technology (Invitrogen). Plasmids were verified by direct DNA sequencing.

### Antibodies

Rabbit polyclonal antibodies against Src (#2109, 1:5000), phosphorylated (p)Src (#2101, 1:1000), pAKT (#9271, 1:1000), AKT (#9272, 1:1000), ERK (#9102, 1:1000), SYK (#2712, 1:1000), FAK (#3285, 1:1000), pTyr (P-Tyr-1000) (#8954, 1:2000), cleaved caspase-3 (#9664, 1:1000), cleaved PARP (#9541, 1:1000), PARP (#9542, 1:1000), cleaved caspase-9 (#9661, 1:1000), caspase-9 (#9508, 1:1000), and HA (#3724, 1:5000) were obtained from Cell Signaling Technologies. Polyclonal IgG (sc-2027), pERK (sc-7383, 1:500), CBL (sc-170, 1:500), SHP2 (sc-280, 1:1000), pMEK1/2 (sc-81503, 1:500), and MEK-1 (sc-6250, 1:500) were obtained from Santa Cruz Biotechnology. p-SHP2(Y542) (ab62322, 1:20,000) was obtained from Abcam. Monoclonal antibodies against Pan-Ras (OP40, 1:500), HA (12CA5, 1:500), and pTyr (4G10) (05–321, 1:1000) were obtained from Boehringer Ingelheim and Millipore, respectively. Monoclonal FLAG-M2 (F1804, 1:2000) and Vinculin (V9264, 1:2000) were obtained from Sigma.

### Chemicals

Compound 11a-1, 6-Hydroxy-3-iodo-1-methyl-2-(3-(2-oxo-2-((4-(thiophen-3-yl)-phenyl)amino)acetamido)phenyl)-1H-indole-5-carboxylic acid was developed and synthesized using a structure-guided and fragment-based library approach^14^. SHP099 was obtained from Novartis. KX2-391 was obtained from Selleck Chemicals. Calf intestinal alkaline phosphatase was obtained from New England Biolabs. EGF and PDGF-BB were obtained from R&D Systems.

### CRISPR/Cas9-mediated gene editing

pLentiCRISPR (49535) was obtained from Addgene, and the following sequences derived from exon 1 of the indicated genes were used to create guides: SHP2 (mouse), 5′-CTGAACCAGTTCAGCCAAAG; SHP2 (human), 5′-GAGACTTCACACTTTCCGTT; and Non Target, 5′-GCGAGGTATTCGGCTCCGCG. The cells were infected with lentivirus as described below.

### Lentiviral production and infection of cell lines

HEK293FT cells (Thermo Fisher Scientific) were transfected with psPAX2, pMDG1.vsvg, and pLentiCRISPR transfer vector. Lentivirus containing supernatant was collected at 72 h post-transfection. Lentiviral supernatant was filtered and applied to the indicated cell lines. MEF and CFPAC1 cells required the addition of 5 μg/ml Polybrene (Millipore). Selection was started 24 h after infection using puromycin (5 μg/ml; Wisent). Monoclonal populations were generated and used for experiments.

### Src knockdown via siRNA

Endogenous Src in *SHP2−/−* MEF cells was silenced using ON-TARGETplus SMARTpool siRNA (Dharmacon) according to the manufacturer’s instructions.

### Immunoprecipitation and immunoblotting

Cells were harvested in EBC lysis buffer (50 mM Tris, pH 8, 120 mM NaCl, 0.5% NP-40) supplemented with protease and phosphatase inhibitors (Roche). Lysates were immunoprecipitated using the indicated antibodies along with protein A-Sepharose (Repligen). Bound proteins were washed five times in NETN buffer (20 mM Tris, pH 8, 100 mM NaCl, 1 mM EDTA, 0.5% NP-40), eluted by boiling in sample buffer, and resolved by SDS-PAGE. Proteins were electrotransferred onto polyvinylidene difluoride membrane (Bio-Rad), blocked, and probed with the indicated antibodies.

### Cellular RAS activity assay

RAS activity was assessed using the RAS activation assay kit from Millipore (17–218). Briefly, RAS-GTP from various treated lysates was pulled-down using an agarose-bound glutathione *S*-transferase (GST) fusion protein corresponding to human RBD of RAF-1. The presence of RAS-GTP was detected by western blotting using an anti-RAS antibody (Millipore # 05–516, 1:2000).

### Cell proliferation assay

Equal numbers of cells were plated in quadruplicate in 96-well plates in the presence or absence of the indicated inhibitors and cellular proliferation was assessed using Alamar Blue proliferation assay as per the manufacturer’s instructions (Invitrogen).

### Recombinant protein expression

The kinase domain from Src was produced recombinantly in *Escherichia coli*^63^. Briefly, DNA corresponding to Src (residues 254–536) was subcloned into pET46 Ek/LIC. Additionally, DNA corresponding to full-length *Yersinia pestis* YopH phosphatase was subcloned into pRSF Ek/LIC. In both constructs, a thrombin protease site was introduced after the vector encoded N-terminal 6-histidine purification tag. Soluble expression of Src was achieved by co-expression of YopH in Rosetta-2 (DE3) *E. coli* cells. Cell cultures were grown to an OD_600_ = 0.8 at 37 °C and recombinant protein expression was induced with a final concentration of 0.5 mM IPTG for 20 h at 18 °C. Pelleted cells were resuspended in 50 mM Tris-HCl pH 8.0, 500 mM NaCl, 25 mM imidazole, and 5% (v/v) glycerol. *E. coli* cells were lysed using a hydraulic cell disruption system (Constant Systems) and recombinant protein was purified by standard Ni-NTA affinity chromatography. Pooled elutions containing both Src and YopH were then dialyzed overnight against 40 volumes of buffer containing 20 mM Tris-HCl pH 8.0, 100 mM NaCl, 5% (v/v) glycerol, and 1 mM dithiothreitol (DTT). Following overnight dialysis, the protein solution was applied onto an anion exchange column (Mono Q 5/50 GL) equilibrated with 20 mM Tris-HCl pH 8.0, 5% (v/v) glycerol, and 1 mM DTT (Buffer A). Protein bound to the anion exchange column was eluted with a 100 CV gradient of 0–50% 20 mM Tris-HCl pH 8.0, 1 M NaCl, 5% (v/v) glycerol, and 1 mM DTT (Buffer B). Anion exchange was sufficient for the separation of recombinant Src kinase from the YopH phosphatase. The elutions containing Src kinase were pooled and further purified by size exclusion chromatography using a custom Superdex-200 10/300 prep grade column equilibrated in 50 mM Tris-HCl, pH 8.0, 100 mM NaCl, 5% (v/v) glycerol, and 1 mM DTT. All purification steps were carried out at 4 °C. Protein concentration was determined by absorbance at *λ* = 280 nm, and purity was confirmed by SDS-PAGE and MS.

The catalytic regions of Son of Sevenless SOS^cat^ (residues 564–1049) and GAP domain of human RASA1 (residues 715–1074) were subcloned into pET15b (Novagen/EMD Biosciences). RBD of BRAF (residues 150–233) fused with N-terminal GST tag was subcloned into pGEX-4T2 (HE Healthcare). The proteins were expressed in *E. coli* BL21 (DE3). GST-RBD was purified using Glutathione Sepharose 4B (GE Healthcare) followed by gel filtration chromatography (Superdex™ S200, GE Healthcare). His-tagged SOS^cat^ and RASA1 GAP domain were purified using Ni-NTA (Qiagen). The His tag was cleaved by thrombin following elution with 250 mM imidazole, and final purification was achieved by gel filtration chromatography (Superdex™ S75, GE Healthcare)^64^.

### Purification of isotopically labeled (^15^N) KRAS

A synthetic gene (Genscript) encoding the GTPase domain of KRAS4B (residues 1–173, with C118S mutation) was cloned with a thrombin-cleavable His-tag into pET-28 and transformed into *E. coli* BL21 (DE3). The bacteria were cultured in minimal M9 medium supplemented with 1 g/l ^15^N ammonium chloride at 37 °C until the O.D. 600 reached 0.6, then induced with 0.2 mM IPTG (isopropyl β-D-1-thiogalactopyranoside) at 16 °C overnight. The cells were harvested, resuspended in lysis buffer (50 mM Tris, 150 mM NaCl, 0.1% NP-40, 10% Glycerol, 10 mM Imidazole, 5 mM MgCl_2_, 1 mM phenylmethylsulfonyl fluoride, 10 mM β-mercaptoethanol and lysozyme at pH 8.0), and lysed by sonication. The protein was purified using Ni-NTA resin followed by gel filtration (Superdex™ S75, GE Healthcare) in a buffer containing 20 mM HEPES, 100 mM NaCl, 5 mM MgCl_2_, and 2 mM tris(2-carboxyethyl)phosphine (TCEP), pH 7.4. KRAS copurifies with *E. coli*–derived guanosine nucleotide, and upon completion of purification, the WT protein is loaded with GDP. KRAS G12V was purified in the same manner; however, it comprises a mixture of GDP- and GTP-bound protein due to its impaired GTP hydrolysis, thus it was incubated for several days to allow GTP hydrolysis to proceed to completion.

### In vitro kinase assay

Purified recombinant human KRAS WT was incubated with purified recombinant active His–Src in 100 μl of kinase buffer [50 mM HEPES (pH7.5), 10 mM MgCl_2_, 1 mM EGTA, 0.01% Brij-35, 200 μM ATP] for 1 h at room temperature. Thereafter, 1 ml of binding buffer [50 mM Tris (pH 8), 120 mM NaCl, 0.1% Nonidet P-40, 5% (v/v) glycerol] was added along with the indicated antibodies and protein A-Sepharose (Repligen). Bound proteins were washed five times in binding buffer before elution by boiling in sample buffer and resolved by SDS-PAGE.

### Real-time NMR GTPase assay

To prepare tyrosyl-phosphorylated KRAS for GTPase assays, a sample of ^15^N-KRAS was confirmed to be GDP-loaded by collecting a ^1^H-^15^N HSQC spectrum, then incubated with a catalytic amount of recombinant Src (i.e., 1:125 molar ratio) in the presence of 2 mM ATP, 1 mM activated sodium vanadate, 2 mM imidazole, 1 mM sodium fluoride, and 1.15 mM sodium molybdate. After confirming completion of the phosphorylation reaction by collection of a ^1^H-^15^N HSQC spectrum, the Src protein and excess ATP was removed from the KRAS sample by gel filtration chromatography (Superdex™ S75 GE Healthcare). For nucleotide exchange assays, 40 μl of 250 μM GDP-loaded ^15^N-KRAS or phosphorylated ^15^N-KRAS was incubated with 10× molar excess GTPγS (guanosine 5′-[γ-thio]triphosphate tetralithium salt, Sigma-Aldrich) in a 1.7 mm NMR tube. Sequential ^1^H-^15^N HSQC NMR experiments were collected throughout the time course of the exchange reaction using a Bruker 600 MHz Avance III NMR spectrometer equipped with a 1.7 mm cryogenic TCI MicroCryoProbe. Data were processed using NMRPipe and analyzed using the NMRFAM-SPARKY software^65,66^. To calculate the nucleotide exchange rate, the fraction of RAS bound to GDP based on the ratio of the peak intensities of three cross-peaks sensitive to nucleotide exchange were plotted vs. time and fit to an exponential association curve^67^ using GraphPad Prism 4. GEF assays were performed in the same manner, with the addition of catalytic domain of Son of Sevenless (residues 564–1049 SOS^cat^) at a molar ratio of 1:600 to KRAS. To perform KRAS GTP hydrolysis assays, ^15^N-KRAS was loaded with GTP by incubation with a 10-fold excess of GTP in the presence of EDTA, which were removed by gel filtration chromatography (Superdex 75 10/300, GE Healthcare), before collecting sequential HSQC spectra. To perform GAP assays, recombinant GAP domain of RASA1 (a construct known as GAP-334) was added at a 1:3000 molar ratio to 40 µl of 250 µM ^15^N KRAS loaded with GTP. The sample was then placed in a 1.7 mm NMR tube to acquire sequential ^1^H-^15^N HSQC spectra during the GTP hydrolysis reaction. The NMR data were processed and analyzed in a manner similar to that described above for nucleotide exchange.

### Mass analysis of intact pKRAS

KRAS and pKRAS samples in 20 mM HEPES, 100 mM NaCl, 5 mM MgCl_2_, 2 mM TCEP, 1 mM activated sodium vanadate, 2 mM imidazole, 1 mM sodium fluoride, and 1.15 mM sodium molybdate at pH 7.4 were diluted 1 to 5 with 20 mM Tris-Base, 5 mM MgCl2, 2 mM TCEP, pH 5.5 to obtain a final concentration of 50 µM. The accurate mass of these samples were obtained using an Agilent 6538 Ultra High Definition (UHD) Quadrupole time-of-flight mass spectrometer run in positive mode with electrospray ionization.

### Ion exchange chromatography

The mono-, di-, and un-phosphorylated forms of KRAS were separated by anion exchange chromatography using a Mono Q 5/50 GL column run with 20 mM HEPES pH 7.0, 5 mM MgCl_2_, and 1 mM TCEP (Buffer A) and 20 mM HEPES pH 7.0, 5 mM MgCl_2_, 1 mM TCEP, and 1 M NaCl (Buffer B). The separation was achieved with an 80 column volume gradient of 0–40% buffer B. Both the wash buffer and elution buffer contained phosphatase inhibitors (1 mM activated sodium vanadate, 2 mM imidazole, 1 mM sodium fluoride, and 1.15 mM sodium molybdate) to stabilize the phosphorylated forms of KRAS.

### Liquid chromatography and MS

Kinase assay samples were reduced with DTT (5 mM) and alkylated with iodoacetamide (10 mM) followed by overnight digestion with trypsin (10 μg/ml in 50 mM NH_4_HCO_3_) at 37 °C. The resulting peptides were de-salted using reversed-phase C18 columns and lyophilized in a vacuum centrifuge. Samples were reconstituted in 0.1% HCOOH and analyzed by LC-MS. Using an EASY-nLC 1000 pump, samples were loaded in-line on an Acclaim PepMap^TM^ 100 nanoViper pre-column (75 μm×2 cm, 3 μm) and resolved on an Acclaim PepMap^TM^ RSLC nanoViper analytical column (75 μm×50 cm, 3 μm) over a 120-min acetonitrile gradient (0–40%) in a 0.1% HCOOH mobile phase. Positive-mode electrospray ionization was applied and ions were analyzed by MS using a Q-Exactive HF instrument set to perform MS/MS HCD fragmentation scans on up to the 20 most intense ions (minimum ion count of 1000 for activation) from an MS parent ion scan (390–1800 *m*/*z* range; 60,000 full-width half-maximum resolution @ 200 *m*/*z*). Fragmented ions were placed on a dynamic exclusion list for 5 s. Acquired raw files were converted to the.mgf format using Proteowizard (v3.0.10800), then searched using X!Tandem (v2013.06.15.1) against Human RefSeq Version 45 (36,113 entries). Search parameters specified a parent ion tolerance of 15 ppm and a fragment ion tolerance of 0.4 Da, with one missed cleavage allowed for trypsin. Carbamidomethylation [C] was set as a fixed modification and oxidation [M], deamidation (N,Q), acetylation (Protein N-term), and phosphorylation (STY) were allowed as variable modifications. All data are publicly available and have been uploaded to the MassIVE archive (https://massive.ucsd.edu).

### Biolayer interferometry

The affinity of KRAS and pKRAS was determined using an Octet RED-384 biolayer interferometry instrument equipped with the Octet Data Acquisition 9.0.0.37 and FortéBio Data analysis software (Pall). The assay was performed using 96-well plates at 25 °C with 1000 rpm agitation in a buffer comprising 20 mM HEPES, 100 mM NaCl, 5 mM MgCl_2_, and 2 mM TCEP supplemented with 1% BSA and 0.005% Tween-20 to minimize nonspecific binding and cocktail of phosphatase inhibitors (1 mM activated sodium vanadate, 2 mM imidazole, 1 mM sodium fluoride, and 1.15 mM sodium molybdate). GST-tagged BRAF-RBD (residues 150–233) (2.5 μg/ml) was immobilized to anti-GST-conjugated biosensors (Pall FortéBio), which were then dipped into wells containing increasing concentrations of KRAS for 30 s, followed by a dissociation step in buffer. A sensor with immobilized BRAF-RBD was dipped into buffer only to subtract instrument drift. The data were analyzed and fitted for global analysis to obtain *K*_D_ values assuming one-to-one stoichiometry.

### 3D spheroid assay

In vitro 3D spheroid culture was performed using an Ultra-Low Attachment surface coating 96-well spheroid microplate (Corning). Briefly, equal numbers of cells were plated in 96-well spheroid microplates and cultured in the presence or absence of the indicated inhibitors. Spheroid viability was determined using the CellTiter-Glo 3D Cell viability assay according to the protocol provided by the manufacturer.

### Organoid cultures

PDAC organoid models used in this study were generated by the Princess Margaret Living Biobank (PMLB) Organoid core facility (https://www.livingbiobank.ca/) from PDX tissues as per the published protocols^68^. All organoids used in this study were characterized extensively by PMLB using the following guidelines. Short tandem repeat (STR) profiling was performed on DNA isolated from the organoid culture and the profile was compared with the STR profile of the primary patient material. Second, organoid culture was embedded and histopathological analysis was performed to assess the morphological features. Immunohistochemistry was performed using antibodies that are clinically relevant to the organ site. The doubling rate of each organoid was determined using short-term viability assays and Cell Titer Glo. Flow analysis using EPCAM antibodies was also performed to ensure the epithelial origin of the organoids. Finally, mycoplasma test was performed on all the established organoid models.

### Organoid drug screening

Organoids were dissociated to single cells and seeded on top of a thin layer of Matrigel in 384-well plate (3000/well). Next day, 11a-1 or SHP099 was added in a six-point concentration series to wells in triplicate. Cell viability was assessed by ATP quantification using the CellTiter-Glo 3D luminescence-based assay. Viability values were normalized to vehicle control wells. Experiment was repeated three times.

### Tumor xenograft experiments

The University Health Network (UHN) Animal Care Committee approved the animal study protocols. Thirteen male SCID mice aged 4–6 weeks were implanted with treatment-naive tumors generated from pancreatic-derived xenograft model OCIP.343 obtained from the PMLB Core Facility (Toronto, Canada). When tumor size reached 200 mm^3^, the mice were randomized into three treatment groups; control (*n* = 5), SHP099 (*n* = 5), and Trametinib (*n* = 3). SHP099 (100 mg/kg) and Trametinib (1 mg/kg) were administered via oral gavage daily. Tumor growth was measured twice per week until they reached 1.5 cm in diameter. Tumor volume was calculated as follows: length × width^2^ × 0.52. At the end of the experiment, mice were sacrificed and tumor tissues were harvested for either formalin fixation or immediate snap freezing in liquid nitrogen.

### Statistical analyses

Unpaired two-tailed Student's *t* test was used to compare between treatment groups and cell types. All statistical analysis was performed using the GraphPad PRISM 6.0 software unless otherwise stated. *P* value <0.05 was considered statistically significant.

### Reporting summary

Further information on experimental design is available in the Nature Research Reporting Summary linked to this article.

## Supplementary information

Supplementary Information

Peer Review File

Source Data File

Reporting Summary

## Data Availability

Data supporting the findings of this manuscript are available from the corresponding author upon reasonable request. The MS data are publicly available and have been uploaded to the MassIVE archive (https://massive.ucsd.edu). The Source Data underlying Figs. 1f, 3c, d, 5b–e, and 6b and Supplementary Figs. 1a, b, e, f, h, 2b, c, 3a, b, and 7a, b are provided as a Source Data file.
