# Supplementary material for: Tyrosyl phosphorylation of KRAS stalls GTPase cycle via alteration of switch I and II conformation
Source: Nat Commun. 2019 Jan 15;10:224. doi: 10.1038/s41467-018-08115-8 (PMC6333830; doi:10.1038/s41467-018-08115-8)

Tyrosyl phosphorylation of KRAS stalls GTPase cycle via alteration of Switch I and II conformation

Kano et al.

Supplementary Information

Supplementary Figure S1

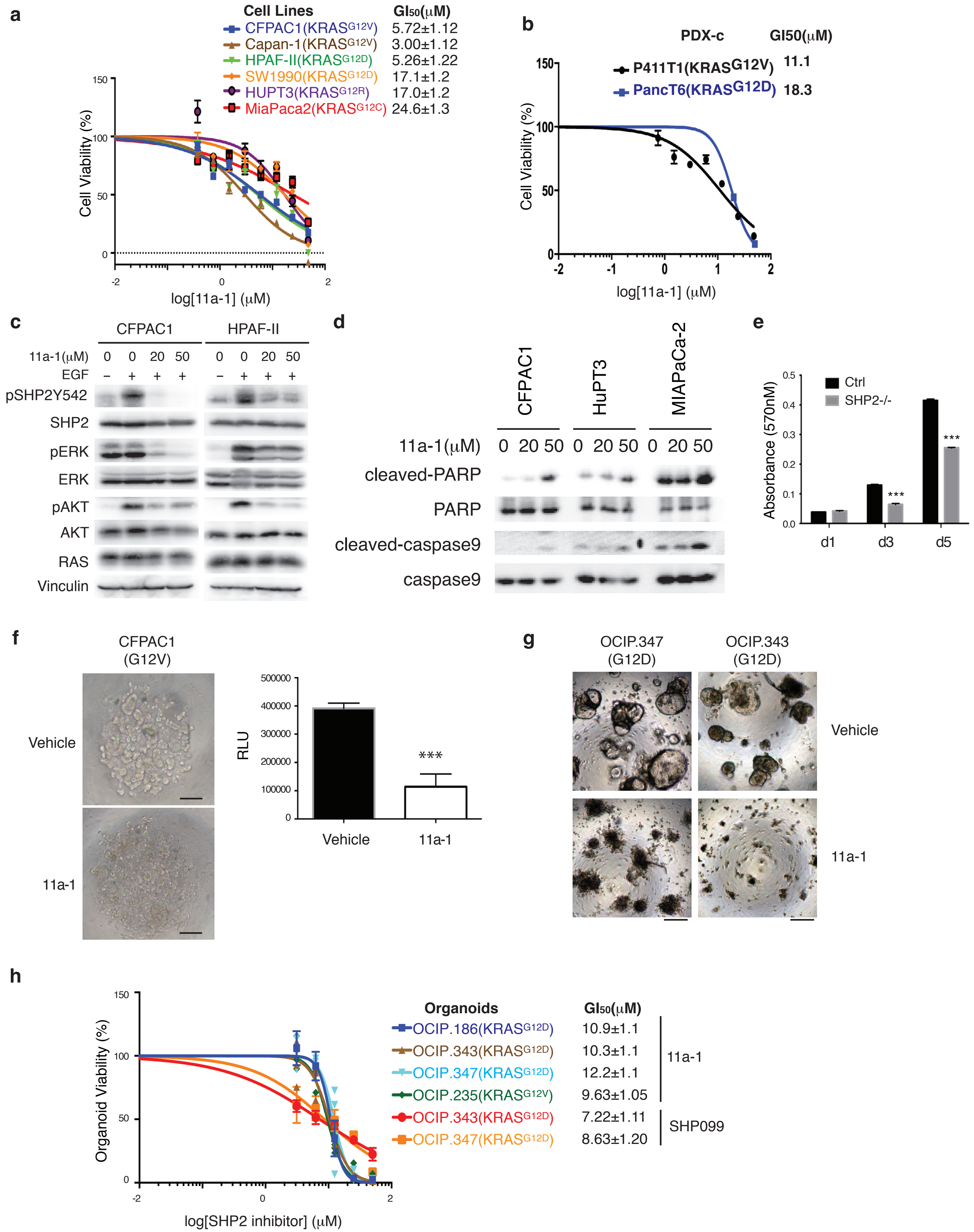

Figure S1. Compound 11a-1 and SHP099 inhibit RAS signaling and promote PDAC cell death in 2D and 3D culture and organoids. (a,b) PDAC cell lines (a) or PDX cells (b) were plated in 96-well plates in sextuplicate, treated with increasing concentrations of 11a-1 for 48 h and then cell viability was determined using alamarBlue. Data represent mean ± s.e.m. of three independent experiments performed in sextuplicate. GI50 values were determined using GraphPad Prism 6.0. (c) PDAC cell lines were serum starved and pretreated with the indicated concentrations of 11a-1 for 5h and then treated with (+) or without (-) 10ng/ml of EGF. Equal amounts of lysates were resolved on SDS-PAGE and immunoblotted with the indicated antibodies. (d) PDAC cell lines were treated with the indicated concentrations of 11a-1 for 24 h. Equal amounts of lysates were resolved on SDS-PAGE and immunoblotted with the indicated antibodies. (e) Equal numbers of Ctrl or SHP2-/- CFPAC1 cells were plated in 96-well plates in sextuplicate and incubated for 120 h and proliferation was assessed using alamarBlue. Data represent mean ± s.e.m. of three independent experiments performed in sextuplicate. ***P < 0.005 Student's t test compared to control. (f) CFPAC1 cells were seeded at 1000 cells/well in 96-well spheroid microplates. Spheroids were cultured for 48 h and then exposed to vehicle (DMSO) or 50μM of 11a-1. Following 48 h incubation with 11a-1, a 3D cell viability assay was performed. (left) Representative images obtained using a VWR Vista Vision inverted microscope. Scale bar, 100 μm. (right) Data represent mean ± s.e.m. of three independent experiments performed in sextuplicates. ***P < 0.005 Student's t test compared to control. (g) Representative images of PDAC organoids treated with 25μM of 11a-1 or vehicle only. PDAC organoids were dissociated to either single cells or small clumps, resuspended in growth media with 2% Matrigel and plated on top of solidified Matrigel (8,000 cells per 96-well plate). Twenty-four hours later, increasing concentrations of 11a-1 were added to the plate and incubated for 96 h. Scale bar, 200 μm. (h) PDAC organoids treated with the indicated concentrations of 11a-1 or SHP099. Organoids viability was determined using an ATP-based proliferation assay (Cell-Titer Glow, Promega). Data represent mean ± s.e.m. of three independent experiments performed in sextuplicate. GI50 values were determined using GraphPad Prism 6.0.

Supplementary Figure S2

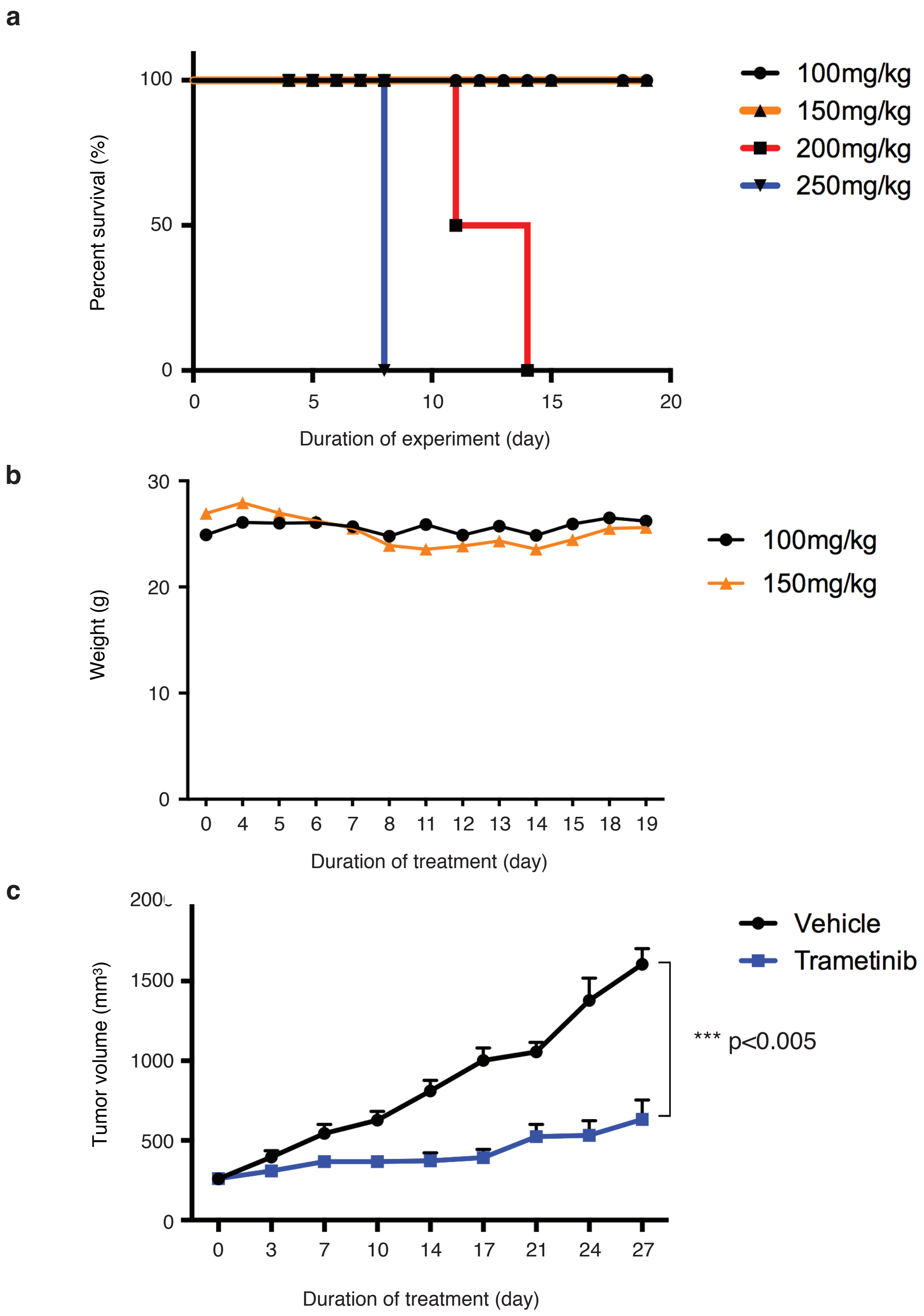

Figure S2. Determination of maximum tolerable drug dose of SHP099 in SCID mice. (a) Survival curves for SCID mice treated daily with SHP099 (100, 150, 200, and 250 mg/kg) for indicated time. Data are plotted as percent survival. N=2 mice/group. (b) Body weight of SCID mice administered with SHP099 (100 and 150 mg/kg) for indicated time. N=2 mice/group. (c) Anti-tumor efficacy of Trametinib (1 mg/kg) administered orally for 28 consecutive days in OCIP.343 xenograt model. Data are shown as mean + s.e.m. N=5 mice/group. ***P < 0.005 Student's t test compared to vehicle.

Supplementary Figure S3

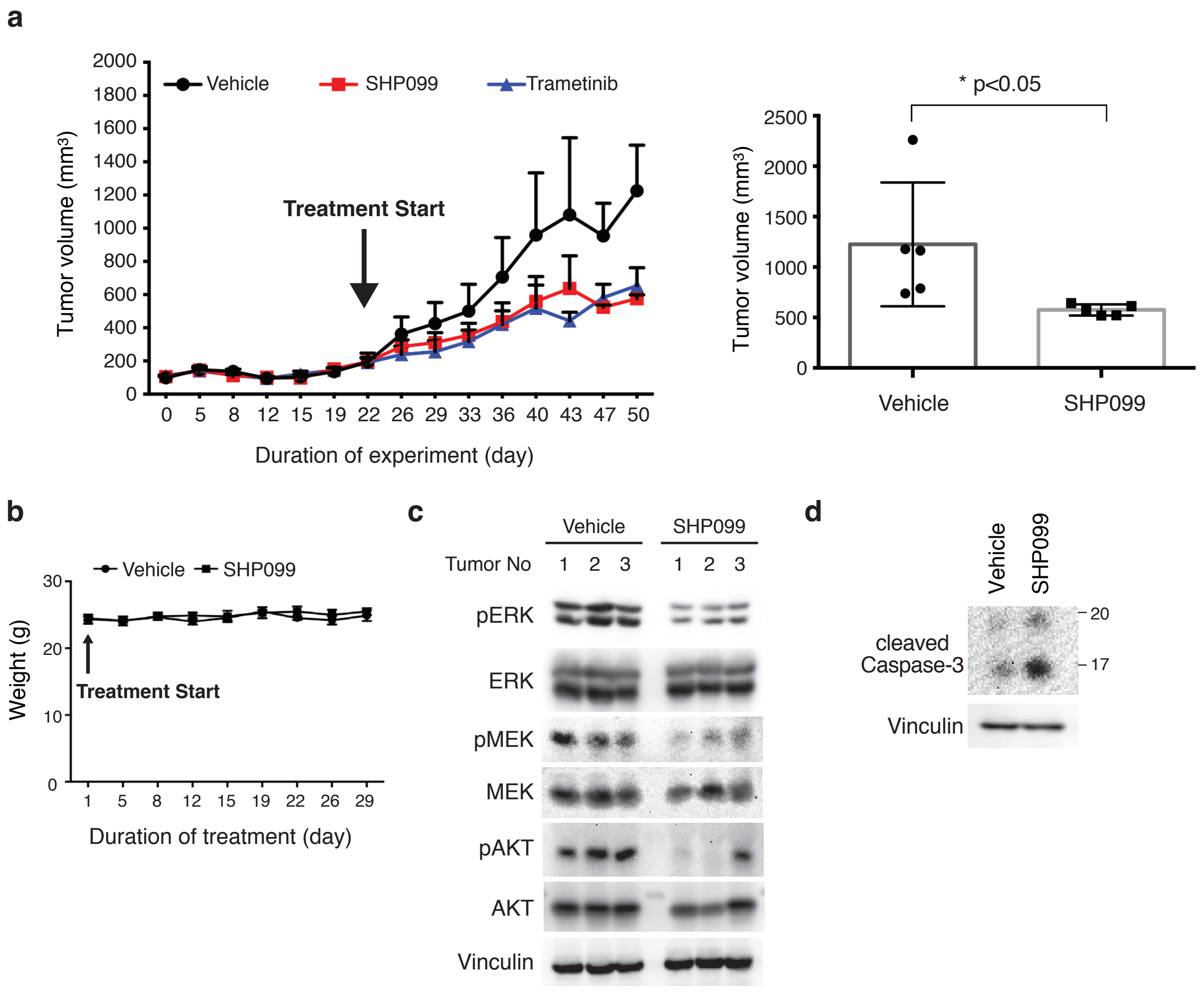

Figure S3. SHP099 suppresses PDAC-PDX tumor growth and dephosphorylation in vivo. (a) Left panel: OCIP.343 subcutaneous xenograft in mice following treatment with vehicle (n=5), SHP099 (100mg/kg, daily, n=5) or Trametinib (1mg/kg, daily, n=3). Data are shown as mean + s.e.m. Right panel: Tumor volume of the indicated treatment groups at the end of experiment. *P < 0.05 Student's t test compared to vehicle. N=5 mice/group. (b) Body weight of mice bearing OCIP.343 subcutaneous xenograft administered with SHP099 or vehicle for 28 consecutive days. Data are shown as mean + s.e.m. N=5 mice/group. (c,d) Equal amounts of OCIP.343 xenograft tumor lysates from the indicated treatment groups were resolved on SDS-PAGE and immunoblotted with the indicated antibodies.

Supplementary Figure S4

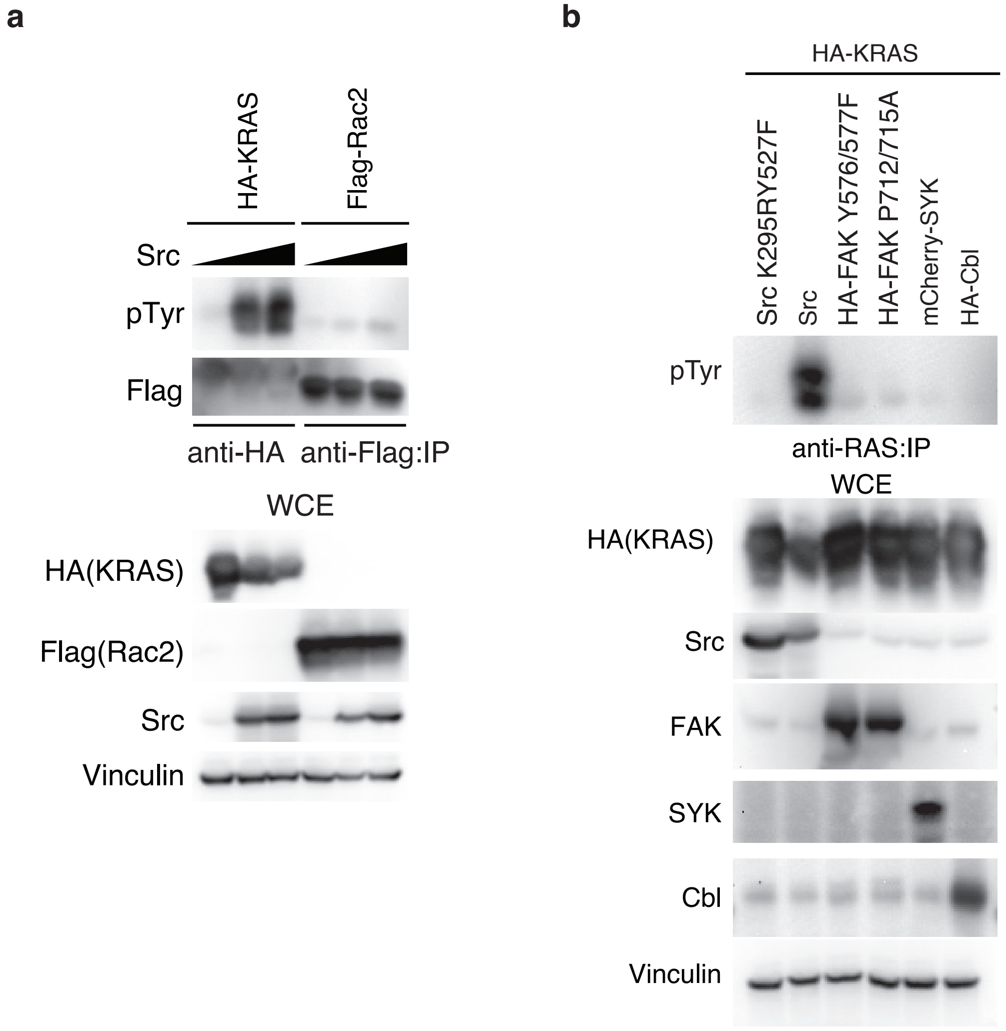

Figure S4. Specificity of Src kinase-mediated tyrosyl phosphorylation of KRAS. (a) HEK293 cells were transfected with KRAS or Rac2 and Src, as indicated plasmids. Cells were lysed, immunoprecipitated and immunoblotted with the indicated antibodies. (b) HEK293 cells were transfected with KRAS together with kinase-dead Src(K295RY527F), Src(WT), kinase-dead HA-FAK(Y576/577F), catalytically active HA-FAK(P712/715A), mCherry-SYK(WT) kinases or HA-CBL(WT) as indicated. Cells were lysed, immunoprecipitated and immunoblotted with the indicated antibodies.

Supplementary Figure S5

a

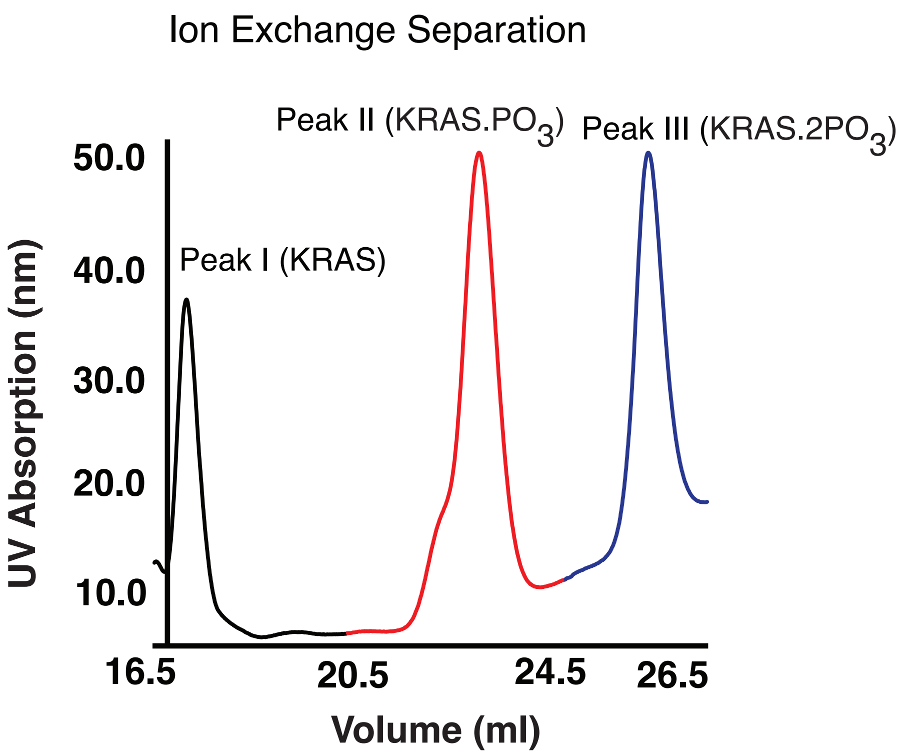

b

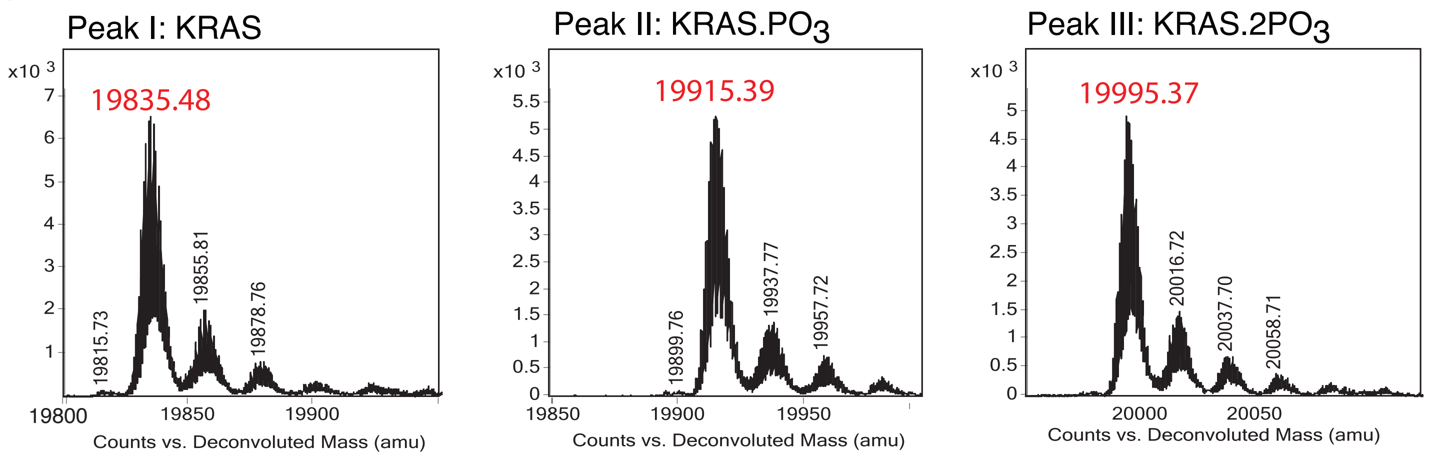

Figure S5. Separation of Src-phosphorylated KRAS into unmodified, mono- and di-phosphorylated forms using anion exchange chromatography. (a) After overnight incubation of KRAS with Src (1: 250 Src:KRAS molar ratio) and 2mM ATP, the unmodified, singly phosphorylated (pKRas.PO3) and doubly phosphorylated (pKRAS.2PO3) forms were separated using anion exchange chromatography A Mono Q 5/50 GL column was run with 20mM HEPES pH 7, 5 mM MgCl2, and 1mM TCEP (Buffer A), and 20mM HEPES pH 7, 5 mM MgCl2, 1mM TCEP, and 1M NaCl (Buffer B) using a gradient of 0 to 40% B over 80 column volumes. Both buffers contain cocktail of phosphatase inhibitors (1mM activated sodium vanadate, 2 mM imidazole, 1 mM sodium fluoride, and 1.15 mM sodium molybdate) (b) Mass spectra of fractions containing umodified (left), mono-phosphorylated (middle) and di-phosphorylated (right) forms of KRAS.

Supplementary Figure S6

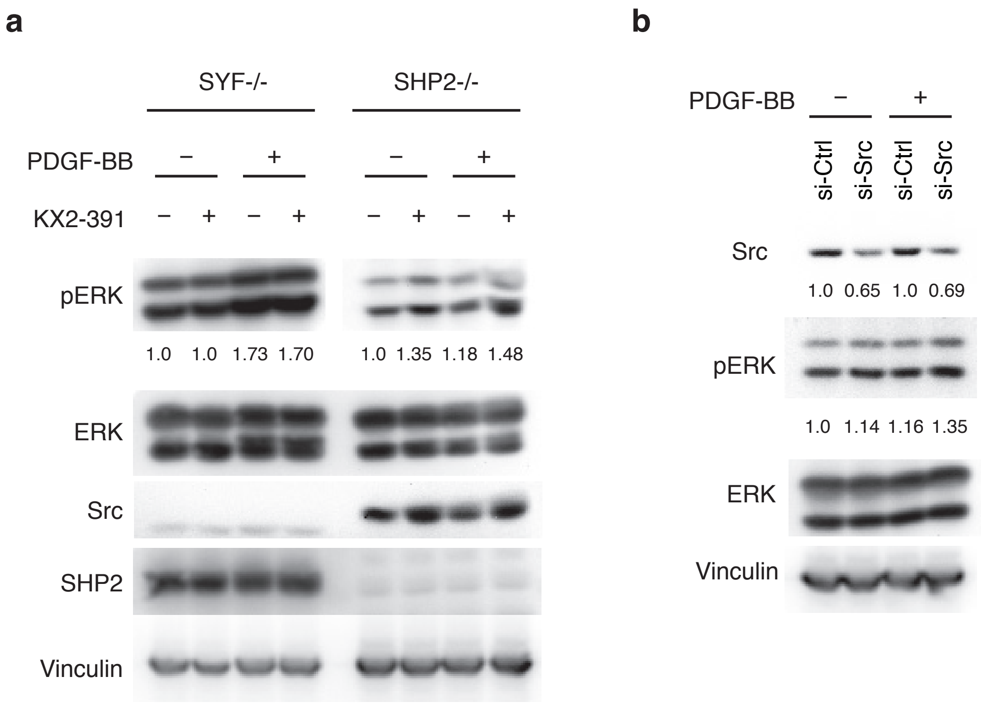

Figure S6. Inhibition of Src enhances ERK signaling in SHP2-/- MEFs.
(a) SYF -/- and SHP2-/- MEFs were serum starved and pretreated with the 500nM of KX2-391 for 2 hr and then treated with (+) or without (-) 20 ng/ml of PDGF-BB for 5 minutes. Equal amounts of lysates were immunoblotted with the indicated antibodies. (b) SHP2-/- MEFs transfected with Src-specific siRNA (si-Src) or a non-targeting scrambled siRNA (si-Ctrl) in combination with the indicated plasmids were treated with (+) or without (-) 20 ng/mL of PDGF-BB for 5 minutes. Cells were then lysed and immunoblotted with indicated antibodies. Numerical values indicate the ratio of densitometrically quantified signals over the control. Densitometric quantification of immunoblots was performed with ImageJ software.

Supplementary Figure S7

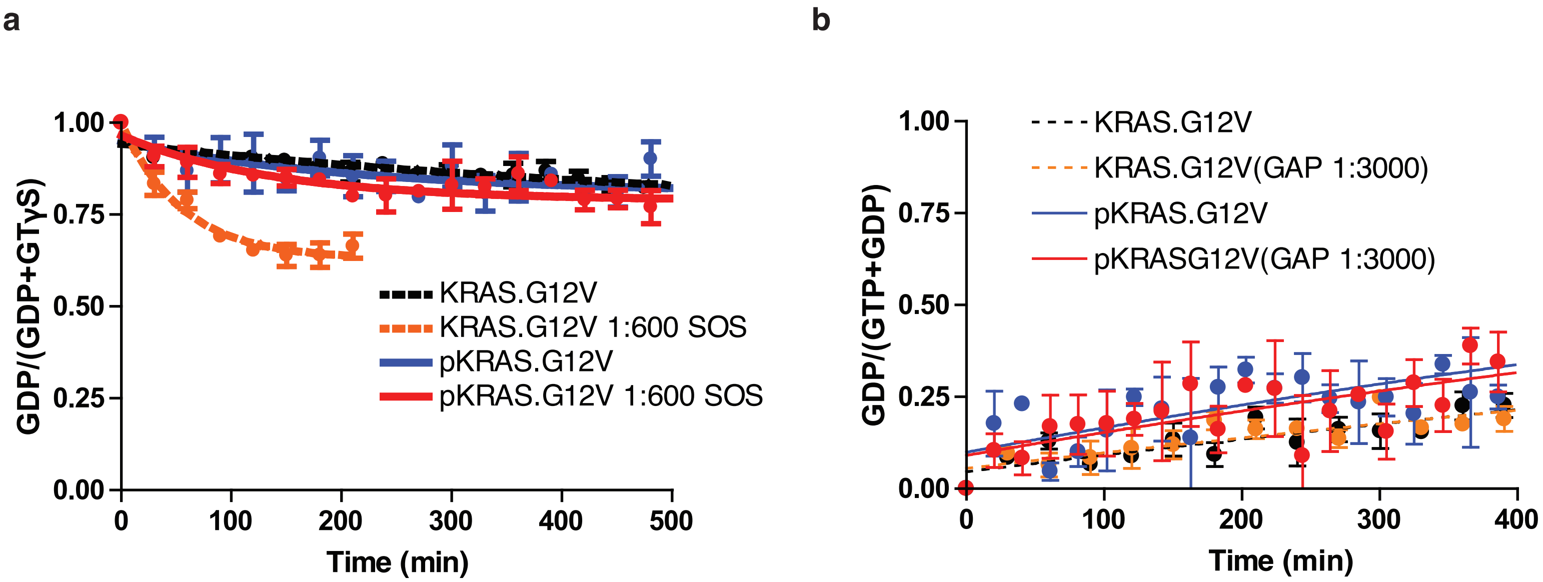

Figure S7. Impact of tyrosyl phosphorylation on the GTPase cycle of KRASG12V. (a) Real-time NMR-derived nucleotide exchange curves for unmodified versus Src-phosphorylated KRASG12V. A 250 μM samples of GDP-loaded 15N KRASG12V (black) or pKRASG12V (blue) was incubated with 10-fold molar excess of GTPγS. Each dot represents the mean fraction of KRAS that is loaded with GDP on the basis of peak intensities [IGDP/ (IGDP+IGTPγS)] from the same three residues of KRASG12V and pKRASG12V. SOScat was added at a ratio of 1:600 to KRASG12V (orange) or pKRASG12V (red). (b) GTP hydrolysis curves illustrating intrinsic and RASA1 GAP domain (1:3000 ratio) assisted GTP hydrolysis for unmodified KRASG12V and pKRASG12V. Each curve was derived from a single representative experiment, which was performed at least twice. Error bars represent the standard deviation of the fraction GDP as reported by three pairs of cross-peaks.

Supplementary Figure S8

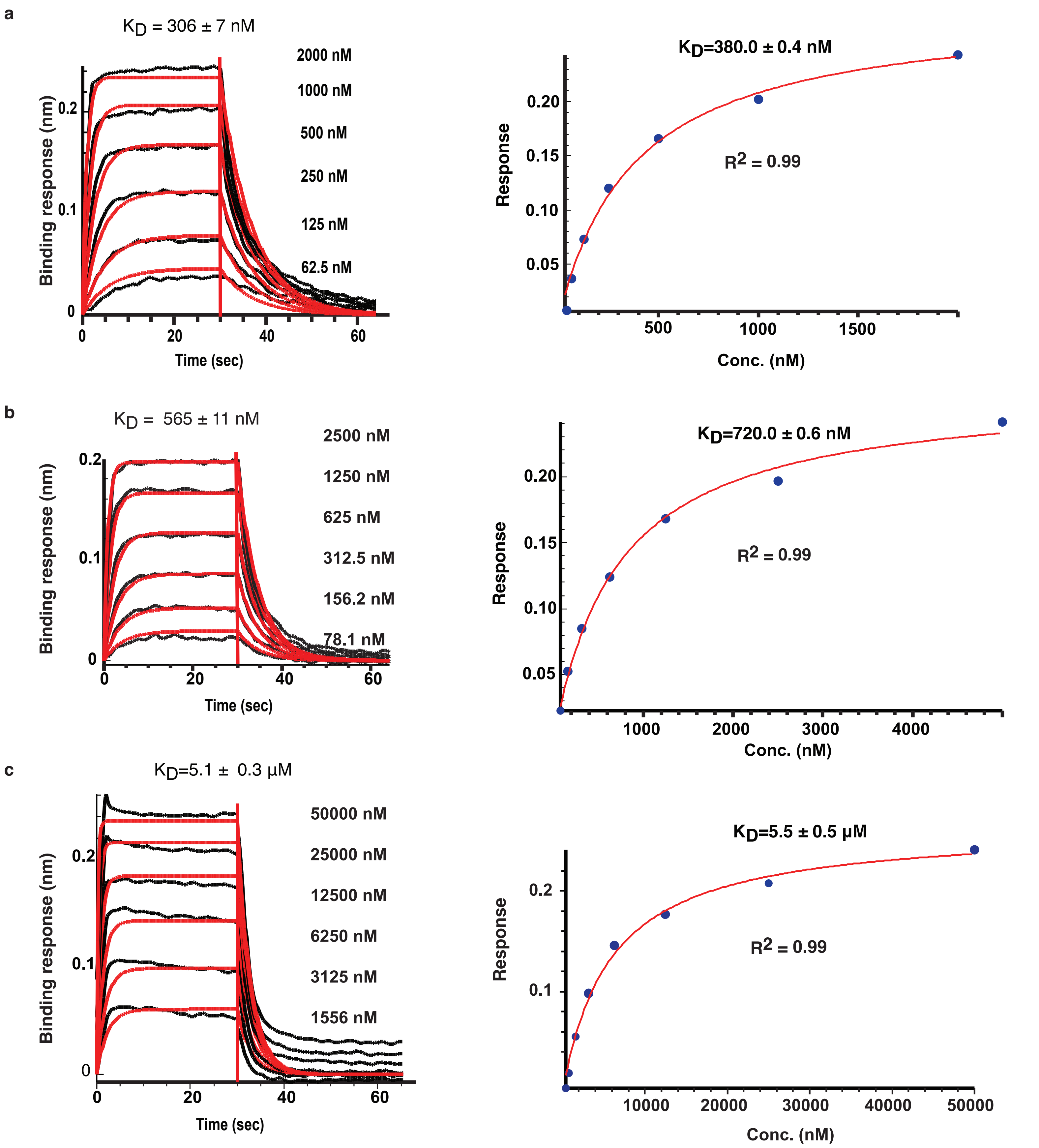

Figure S8. Src-induced phosphorylation impairs KRAS binding to BRAF. Biolayer interferometry (Octet) analyses of binding of KRAS to immobilized BRAF RBD. Unmodified KRAS (a), mono-phosphorylated KRAS (KRAS.PO3) (b), and di-phosphorylated KRAS (KRAS.2PO3) (c) were used at the concentrations indicated. Left panels show binding response versus time (black) and fitted curves (red) with Kd values determined by kinetic analysis. Right panels show maximal response versus KRAS concentration with Kd values determined by steady state analysis.

Supplementary Table S1

a

	KRAS: Intrinsic		KRAS: SOS assisted		KRAS: Intrinsic		pKRAS: SOS assisted	
	Expt_1	Expt_2	Expt_1	Expt_2	Expt_1	Expt_2	Expt_1	Expt_2
Rate of Exchange (x 1000)	2.75 ± 0.61	3.49±0.32	50.05±5.97	57.47±11.19	7.55±1.5	6.99±1.19	21.35±3.71	15.43±2.28

b

	KRAS: Intrinsic		KRAS: GAP assisted		KRAS: Intrinsic		pKRAS: GAP assisted	
	Expt_1	Expt_2	Expt_1	Expt_2	Expt_1	Expt_2	Expt_1	Expt_2
Rate of Hydrolysis (x 1000)	5.459 ± 0.27	6.062 ± 0.41	15.04 ±0.8	19.27±1.3	1.956±0.24	1.886±0.57	2.556±0.24	2.264±0.36

Table S1. Nucleotide exchange (a) and GTP hydrolysis (b) rates of KRAS and pKRAS. See text for details.

Figure 1a

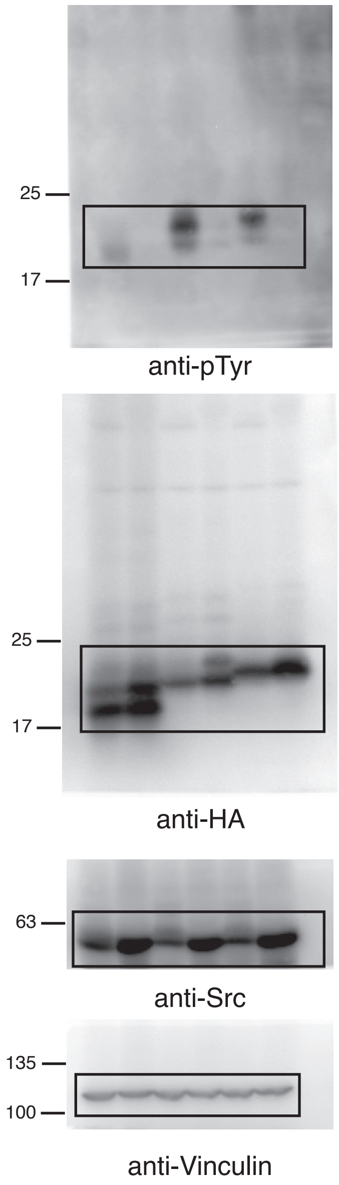

Figure 1b

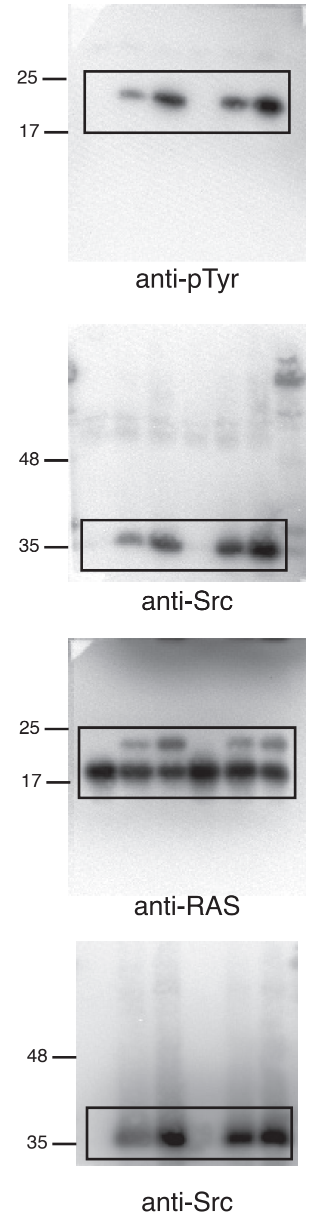

Figure 2e

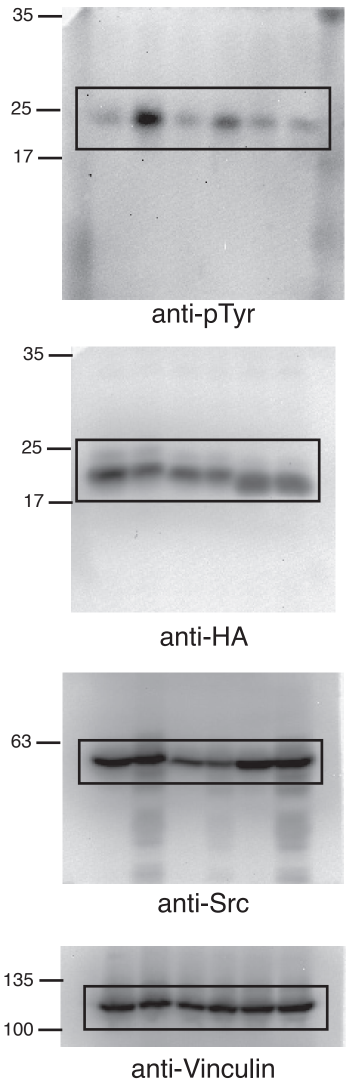

Figure 2f

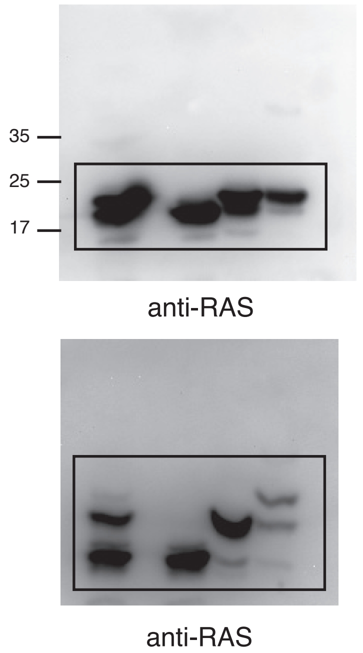

Figure 3a

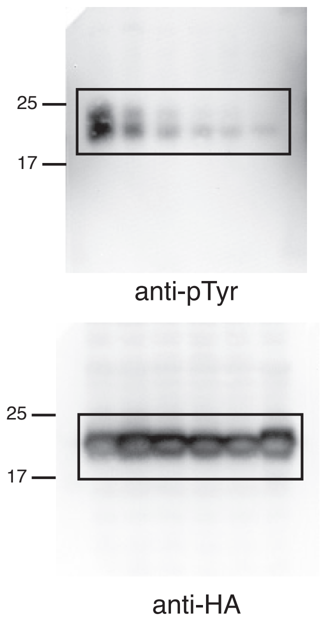

Figure 3a

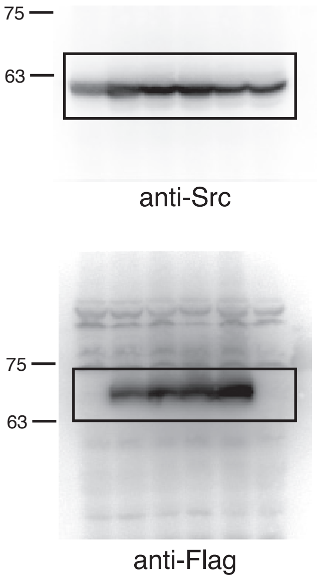

Figure 3a

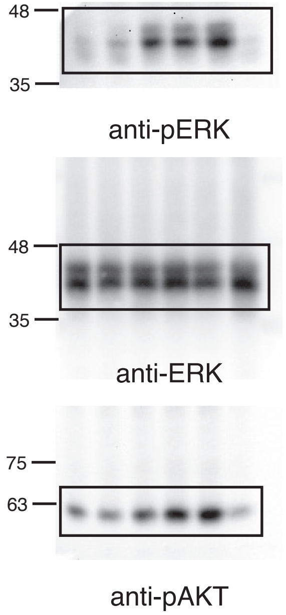

Figure 3a

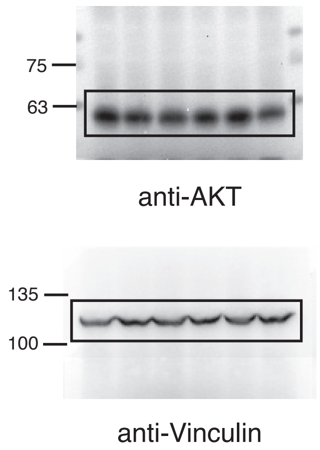

Figure 3b

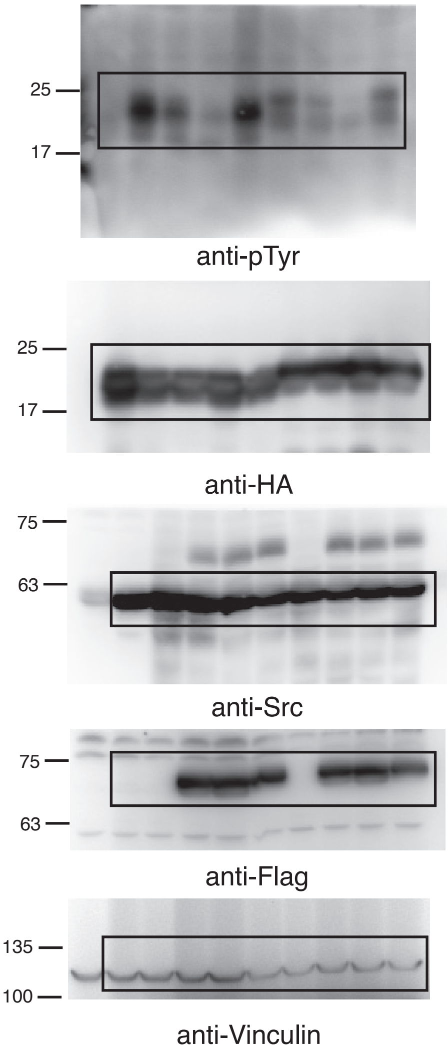

Figure 3f

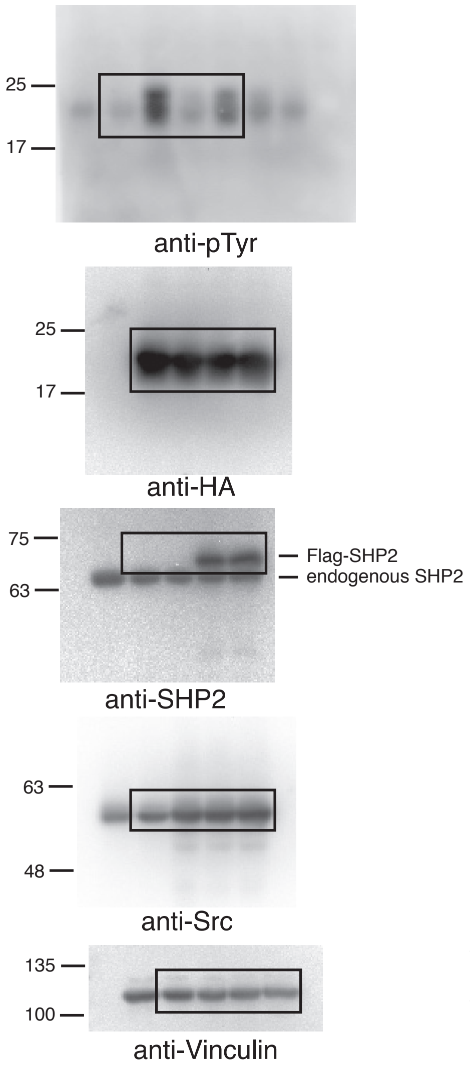

Figure 6d

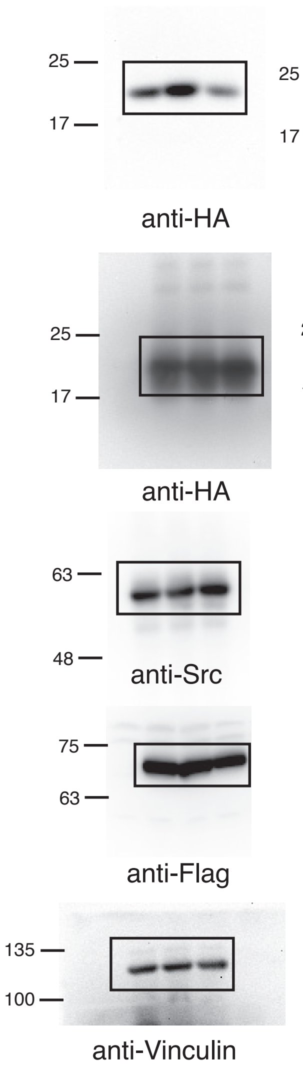

Figure 6d

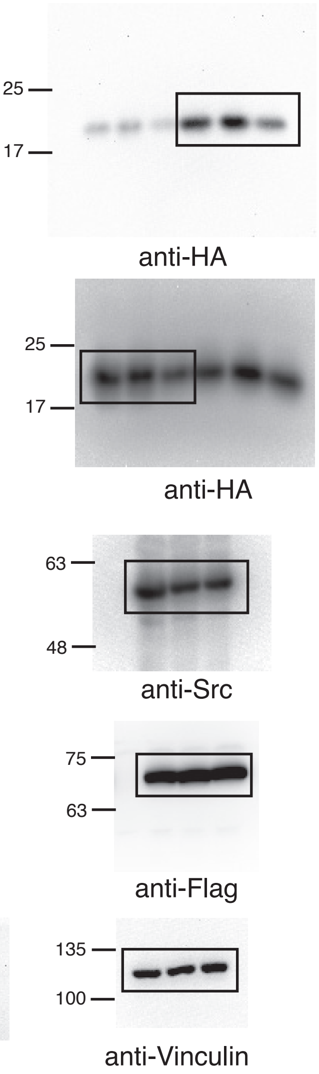

Figure 4a

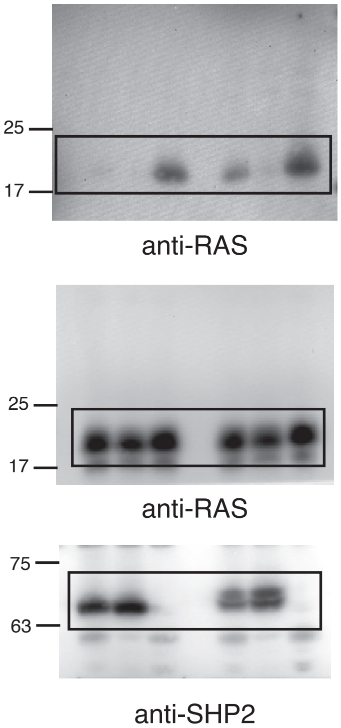

Figure 4a

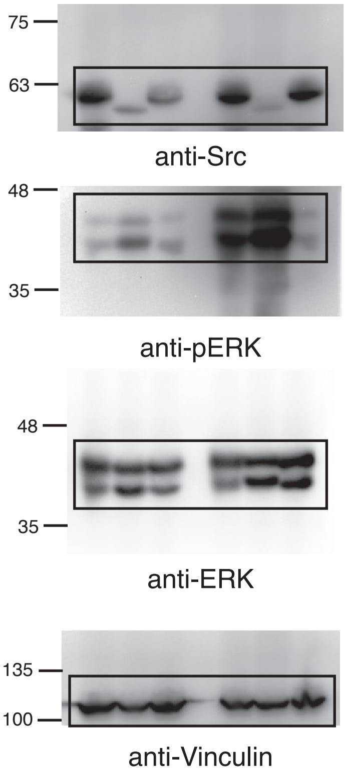

Figure 4a

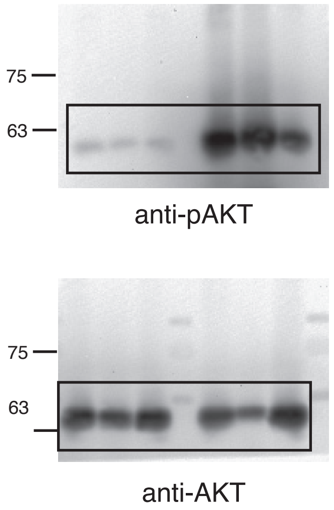

Figure 4b

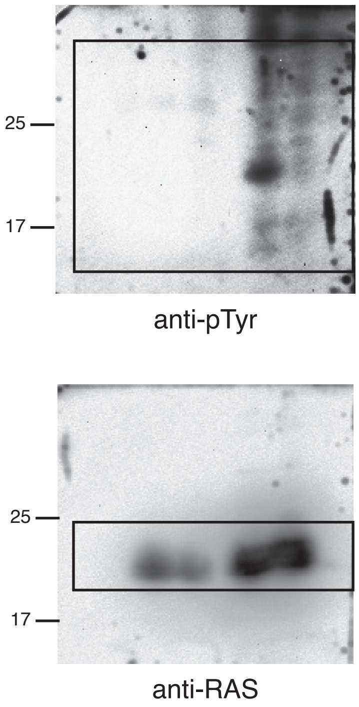

Figure 4b

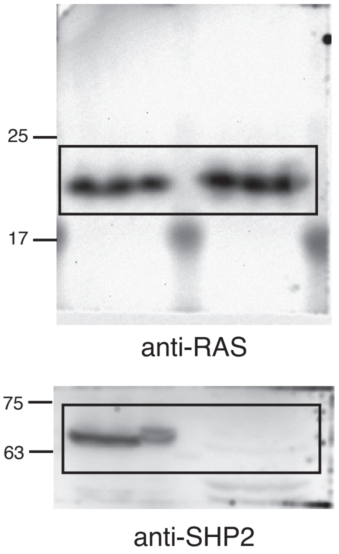

Figure 4b

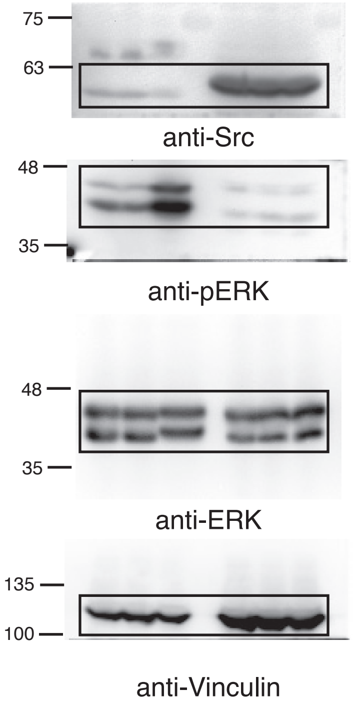

Figure 4c

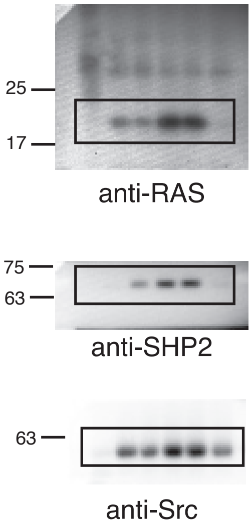

Figure 4c

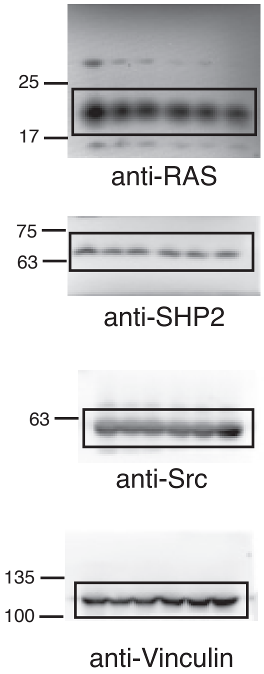

Figure 4d

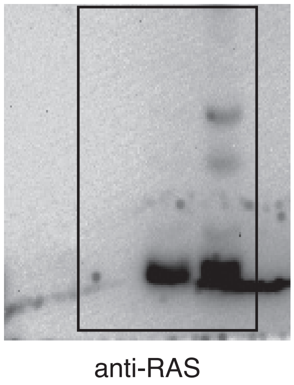

Figure 4e

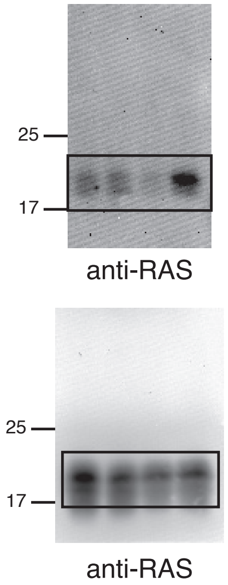

Figure 4e

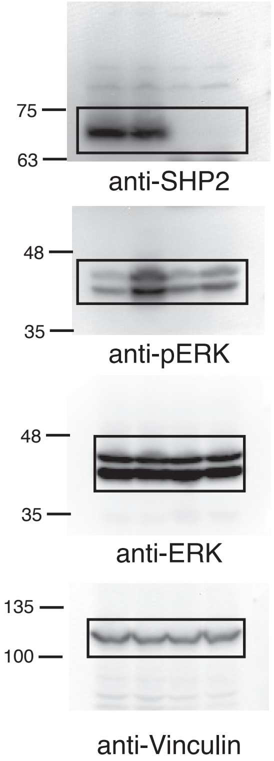

Figure 4f

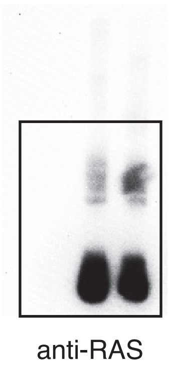

Figure 6a

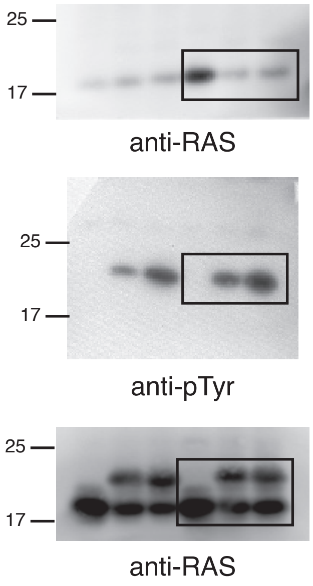

Figure 6a

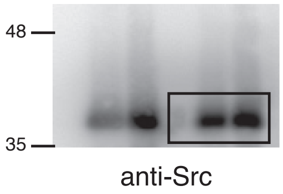

Figure 6c

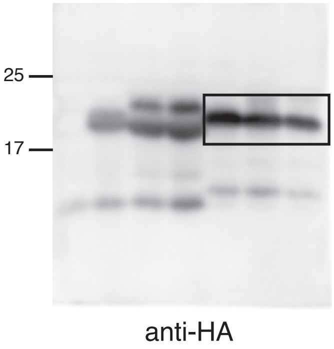

Figure 6c

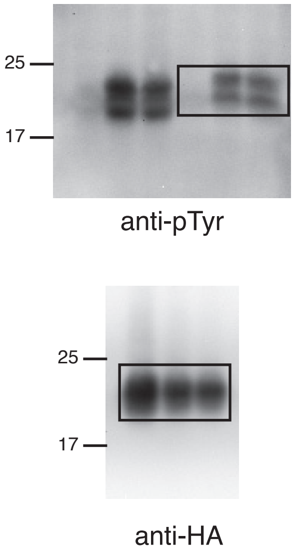

Figure 6c

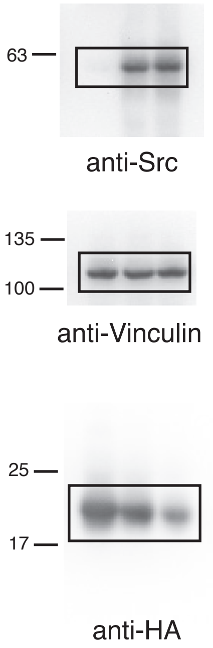

Figure 6c

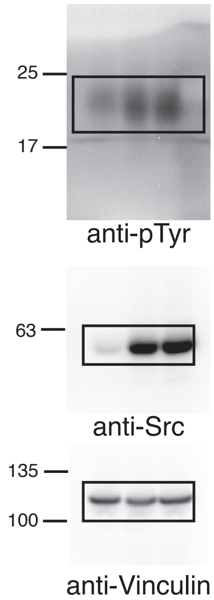

Figure S1c

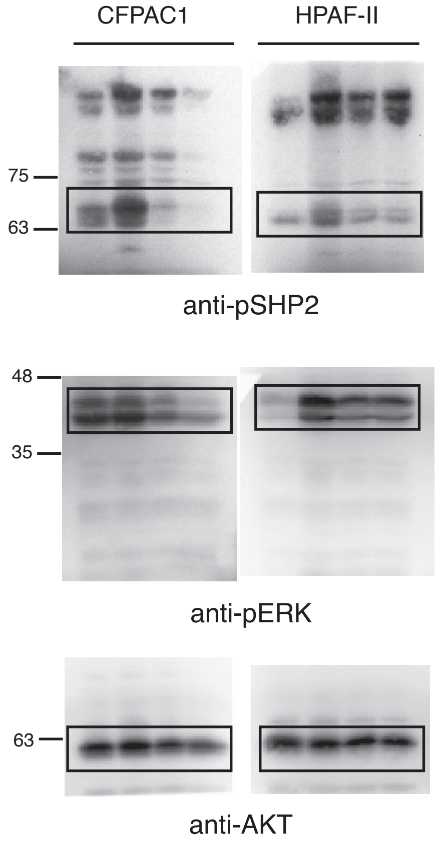

Figure S1c

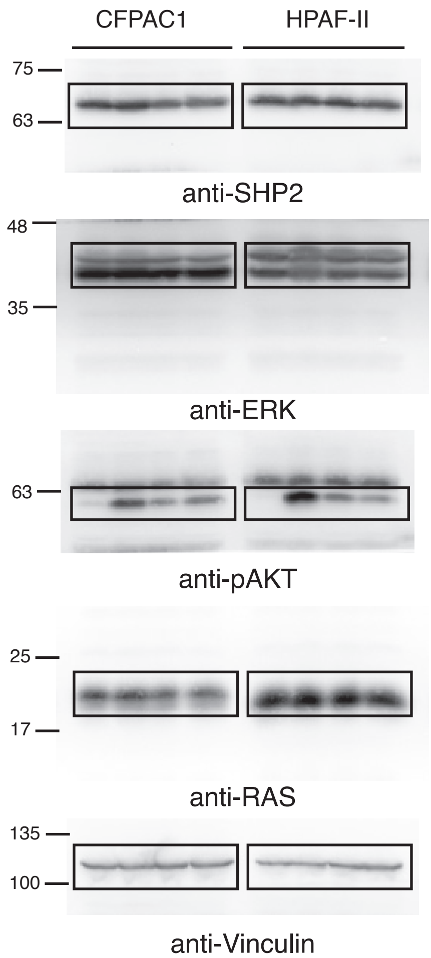

Figure S1d

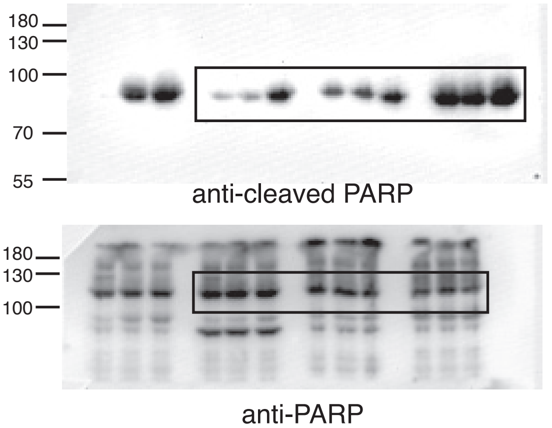

Figure S1d

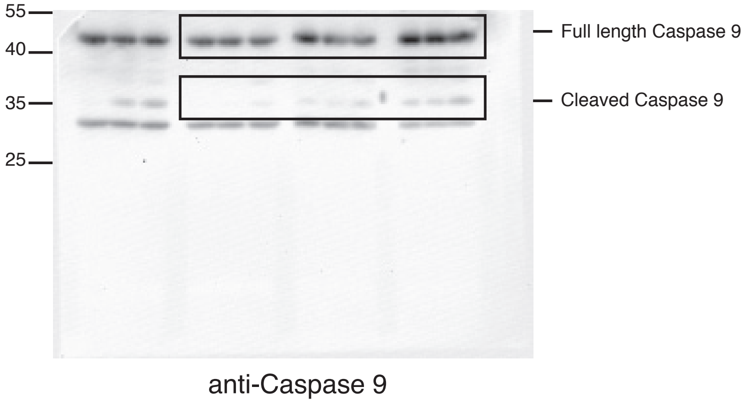

Figure S3c

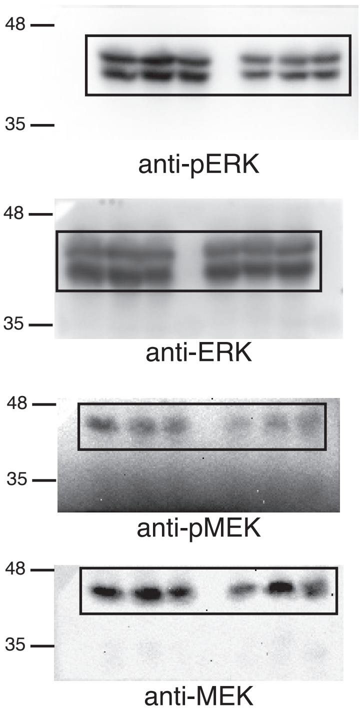

Figure S3c

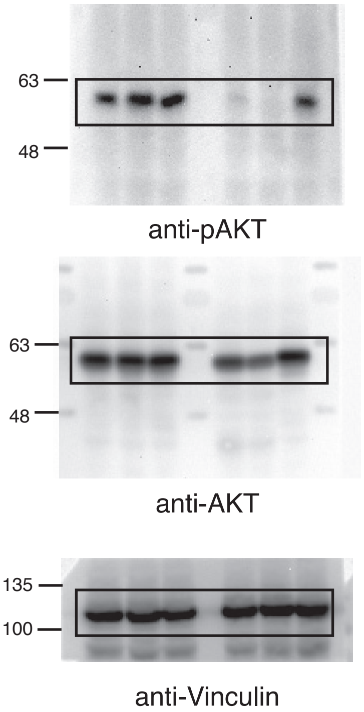

Figure S3d

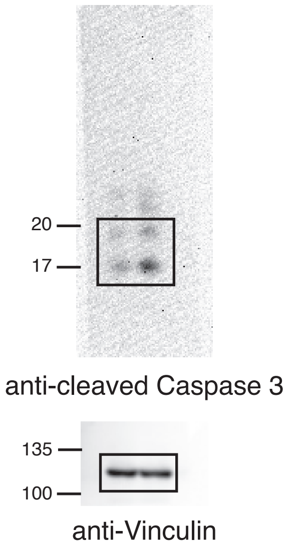

Figure S4a

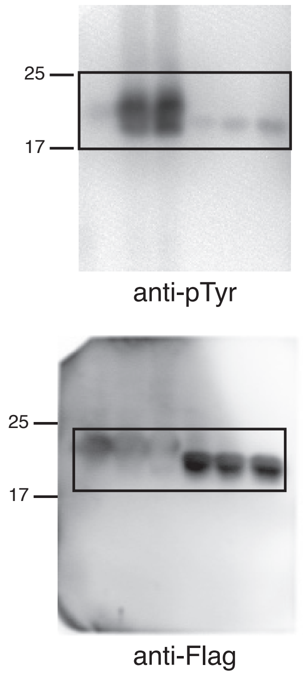

Figure S4a

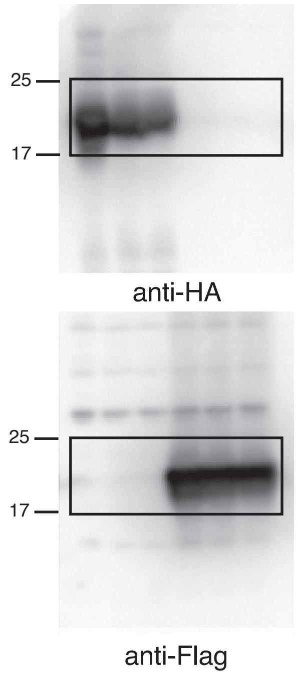

Figure S4a

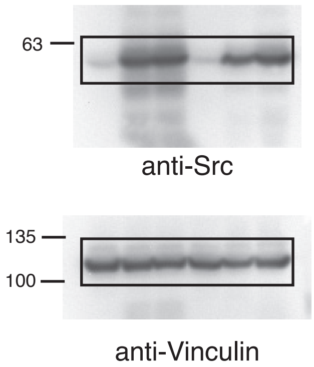

Figure S4b

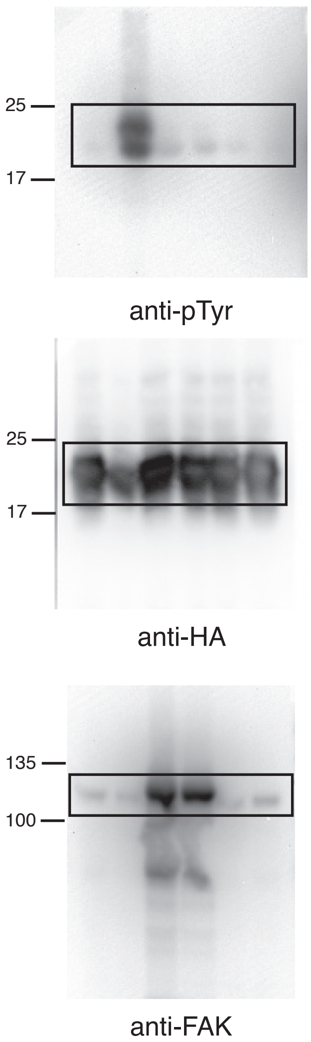

Figure S4b

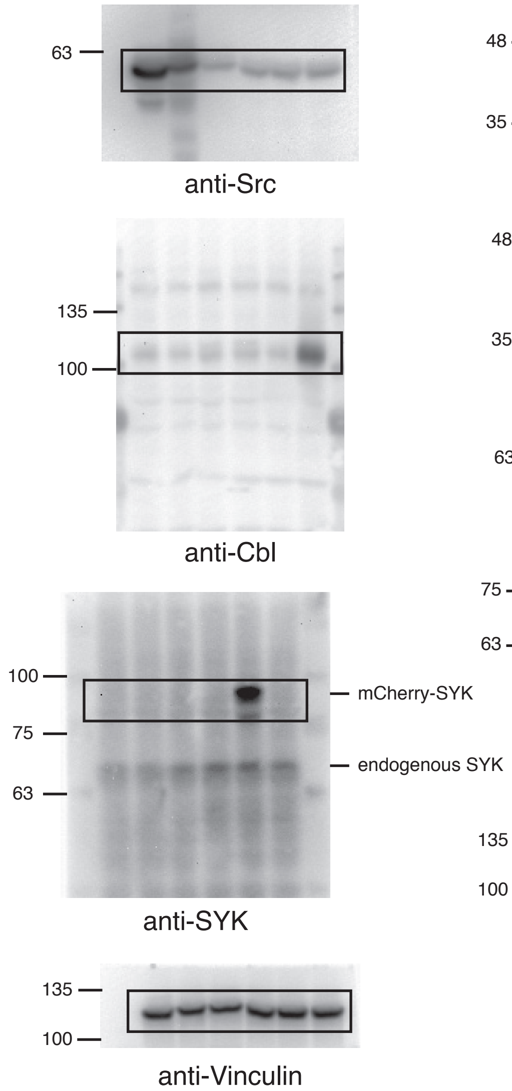

Figure S6a

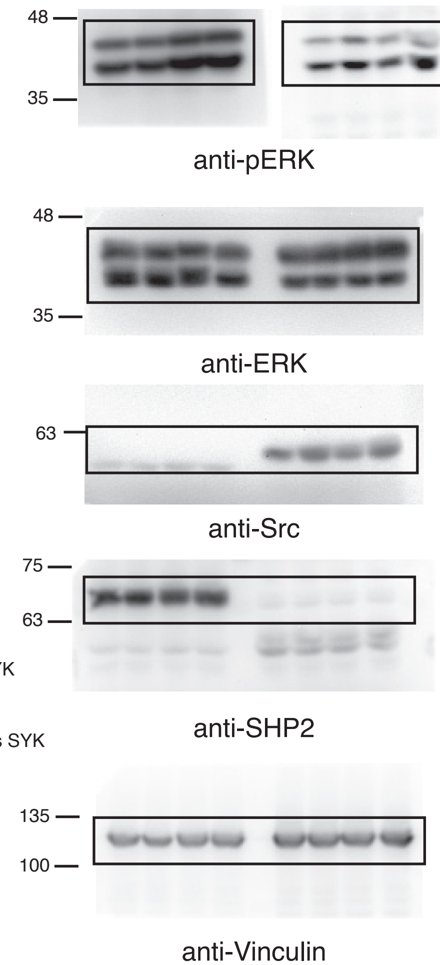

Figure S6b

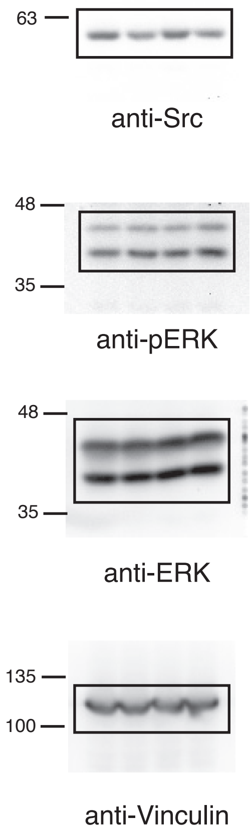

Supplement: Supplementary file 1 — Supplementary Information [file 41467_2018_8115_MOESM1_ESM.pdf]
