# Supplementary material for: Tyrosyl phosphorylation of KRAS stalls GTPase cycle via alteration of switch I and II conformation
Source: Nat Commun. 2019 Jan 15;10:224. doi: 10.1038/s41467-018-08115-8 (PMC6333830; doi:10.1038/s41467-018-08115-8)
Supplement: Supplementary file 4 — Reporting Summary [file 41467_2018_8115_MOESM4_ESM.pdf]

natureresearch

Corresponding author(s): Michael Ohh

Reporting Summary

Nature Research wishes to improve the reproducibility of the work that we publish. This form provides structure for consistency and transparency in reporting. For further information on Nature Research policies, see Authors & Referees and the Editorial Policy Checklist.

Statistical parameters

When statistical analyses are reported, confirm that the following items are present in the relevant location (e.g. figure legend, table legend, main text, or Methods section).

n/a	Confirmed	
☐	☒	The exact sample size (n) for each experimental group/condition, given as a discrete number and unit of measurement
☐	☒	An indication of whether measurements were taken from distinct samples or whether the same sample was measured repeatedly
☐	☒	The statistical test(s) used AND whether they are one- or two-sided<br>*Only common tests should be described solely by name; describe more complex techniques in the Methods section.*
☒	☐	A description of all covariates tested
☐	☒	A description of any assumptions or corrections, such as tests of normality and adjustment for multiple comparisons
☐	☒	A full description of the statistics including central tendency (e.g. means) or other basic estimates (e.g. regression coefficient) AND variation (e.g. standard deviation) or associated estimates of uncertainty (e.g. confidence intervals)
☐	☒	For null hypothesis testing, the test statistic (e.g. F, t, r) with confidence intervals, effect sizes, degrees of freedom and P value noted<br>*Give P values as exact values whenever suitable.*
☒	☐	For Bayesian analysis, information on the choice of priors and Markov chain Monte Carlo settings
☒	☐	For hierarchical and complex designs, identification of the appropriate level for tests and full reporting of outcomes
☒	☐	Estimates of effect sizes (e.g. Cohen's d, Pearson's r), indicating how they were calculated
☐	☒	Clearly defined error bars<br>*State explicitly what error bars represent (e.g. SD, SE, CI)*

Our web collection on statistics for biologists may be useful.

Software and code

Policy information about availability of computer code

Data collection	NMR data was collected using Bruker TopSpin 3.2.<br>BLI data was collected using Octet Data Acquisition 9.0.0.37 (FortéBio).<br>Phosphoproteomics data were collected using Thermo Xcalibur v4.0.
Data analysis	GraphPad PRISM 6.0 software was used to analyze cell/organoid viability and tumour progression data.<br>Densitometric quantification of immunoblots was performed with ImageJ software.<br>NMR data were processed with NMRPipe and analyzed using NMRFAM-SPARKY, and kinetics were analyzed using GraphPad Prism 4.0.<br>BLI data was analyzed with Octet Data Analysis 9.0.0.12 (FortéBio).<br>Mass spectrometry of intact proteins were collected and analyzed using MassHunter Qual B.0.6.0 with Bioconfirm.<br>Phosphoproteomics data were analyzed using Proteowizard (v3.0.10800) and X!Tandem (v2013.06.15.1).

For manuscripts utilizing custom algorithms or software that are central to the research but not yet described in published literature, software must be made available to editors/reviewers upon request. We strongly encourage code deposition in a community repository (e.g. GitHub). See the Nature Research guidelines for submitting code & software for further information.

Data

Policy information about availability of data

All manuscripts must include a data availability statement. This statement should provide the following information, where applicable:

- Accession codes, unique identifiers, or web links for publicly available datasets
- A list of figures that have associated raw data
- A description of any restrictions on data availability

The data from this study are available from the corresponding authors upon reasonable request. Phosphoproteomics data has been deposited in the MassIVE archive (https://massive.ucsd.edu) under the accession number: MSV000083155.

Field-specific reporting

Please select the best fit for your research. If you are not sure, read the appropriate sections before making your selection.

☒ Life sciences ☐ Behavioural & social sciences ☐ Ecological, evolutionary & environmental sciences

For a reference copy of the document with all sections, see nature.com/authors/policies/ReportingSummary-flat.pdf

Life sciences study design

All studies must disclose on these points even when the disclosure is negative.

Sample size	For biochemical assay, at least three separate experiments were done. For PDX experiments, the number of mice per group was determined based on previous experience with the patient-derived tumors used in the study. NMR-based nucleotide exchange and hydrolysis assays were performed at least in duplicate.
Data exclusions	No data was excluded from the studies.
Replication	All attempts at replication were successful.
Randomization	When tumor size reached 200 mm3, the mice were randomized into three treatment groups (control, SHP099 and Trametinib).
Blinding	For analysis of inhibitor studies, each dataset was analyzed independently by two investigators.

Reporting for specific materials, systems and methods

Materials & experimental systems

n/a	Involved in the study	
☒	☐	Unique biological materials
☐	☒	Antibodies
☐	☒	Eukaryotic cell lines
☒	☐	Palaeontology
☐	☒	Animals and other organisms
☒	☐	Human research participants

Methods

n/a	Involved in the study	
☒	☐	ChIP-seq
☒	☐	Flow cytometry
☒	☐	MRI-based neuroimaging

Antibodies

Antibodies used	Rabbit polyclonal antibodies against Src (#2109, 1:5000), phosphorylated (p)Src (#2101, 1:1000), pAKT (#9271, 1:1000), AKT (#9272, 1:1000), ERK (#9102, 1:1000), SYK (#2712, 1:1000), FAK (#3285, 1:1000), pTyr (P-Tyr-1000) (#8954, 1:2000), cleaved caspase-3 (#9664, 1:1000), cleaved PARP (#9541, 1:1000), PARP (#9542, 1:1000), cleaved caspase-9 (#9661, 1:1000), caspase-9 (#9508, 1:1000) and HA (#3724, 1:5000) were obtained from Cell Signaling Technologies. Polyclonal IgG (sc-2027), pERK (sc-7383, 1:500), CBL (sc-170, 1:500), SHP2 (sc-280, 1:1000), pMEK1/2 (sc-81503, 1:500) and MEK-1 (sc-6250, 1:500) were obtained from Santa Cruz Biotechnology. p-SHP2(Y542) (ab62322, 1:20000) was obtained from abcam. Monoclonal antibodies against Pan-Ras (OP40, 1:500), HA (12CA5, 1:500) and pTyr (4G10) (05-321, 1:1000) were obtained from Boehringer Ingelheim and Millipore, respectively. Monoclonal FLAG-M2 (F1804, 1:2000) and Vinculin (V9264, 1:2000) were obtained from Sigma.
Validation	All antibodies used in this study have been validated by the commercial manufacturers from whom they were purchased.

Eukaryotic cell lines

Policy information about cell lines

Cell line source(s)	HEK293, MEF and MEF-SYF(-/-) were obtained from the American Type Culture Collection. BxPC3, CFPAC1, Capan-1, HPAF-II, SW1990, HPAC, Panc-1, HUPT3, MiaPaCa-2, P411T1 and PancT6 cells were kind gifts from Dr. Jen Jen Yeh (University of North Carolina, Chapel Hill). P411T1 and PancT6 were generated from surgical resections. To generate organoids, tumor tissues were obtained by Princess Margaret Living Biobank (PMLB) Organoid core facility from surgical resections performed at University Health Network.
Authentication	All cell lines were verified for purity using STR analysis.
Mycoplasma contamination	All cell lines tested mycoplasma negative.
Commonly misidentified lines<br>(See ICLAC register)	N/A

Animals and other organisms

Policy information about studies involving animals; ARRIVE guidelines recommended for reporting animal research

Laboratory animals	The University Health Network (UHN) Animal Care Committee approved the animal study protocols. Thirteen male SCID mice aged 4-6 weeks were implanted with treatment-naïve tumors generated from pancreatic derived xenograft model OCIP.343 obtained from Princess Margaret Living Biobank (PMLB) Core Facility (Toronto, Canada).
Wild animals	N/A
Field-collected samples	N/A
