# Supplementary material for: Tyrosyl phosphorylation of KRAS stalls GTPase cycle via alteration of switch I and II conformation
Source: Nat Commun. 2019 Jan 15;10:224. doi: 10.1038/s41467-018-08115-8 (PMC6333830; doi:10.1038/s41467-018-08115-8)
Supplement: Supplementary file 2 — Peer Review File [file 41467_2018_8115_MOESM2_ESM.pdf]

Reviewers' Comments:

Reviewer #1:
Remarks to the Author:
In this manuscript, the authors used a range of techniques (from detailed NMR and mass spectrometry to hard-core cell biology and animal models) to unambiguously demonstrate that KRAS is phosphorylated at Tyr32 and Ttyr64 by Src and dephosphorylated by SHP2. Phosphorylation appears to disrupt the KRAS GTPase cycle and signaling while dephosphorylation restores these functions. The authors also show that inhibition of SHP2 reduces oncogenic KRAS signaling. Overall, this a clearly presented and comprehensive piece of work that advances the field of RAS biology. I have only two suggestions (in order of relevance/feasibility):

1) Unlike the role of Src, which has been directly interrogated using NMR, the regulatory functions of SHP2 have not been subjected to a similar analysis. While the cell-biology data is strong, it would be more convincing if the NMR analysis (e.g., measurement of reaction rates) were repeated in a KRAS/Src/SHP2 context and compared with the KRAS/Src data.

2) It would be nice if the authors solved at least one structure of pKRAS, pKRAS/SOS or pKRAS/GAP complexes and replaced the somewhat speculative Fig 5 by an actual data.

Reviewer #2:
Remarks to the Author:
The manuscript by Kano and Gebregiworgis et al. describes the tyrosine phosphorylation of KRAS by SRC and its dephosphorylation by SHP2. The authors report striking biochemical changes associate with phosphorylation, including loss of responsiveness to well-established regulators, as well as a reduced capacity to engage an effector protein in vivo. The findings have a direct bearing on efforts to understand RAS signal transduction in multiple aspects of cell biology. There are also clear implications for the treatment of cancers that are driven by mutant RAS proteins or their upstream activators. As such, this work should be of interest to a wide readership. There are, however, several substantive and stylistic issues that should be addressed prior to a final editorial decision.

Problems that need to be addressed:
Data presentation and interpretation
1. The data in Supplementary Figure S1a are a weak argument for the specificity of the drug 11a-1. The viability curves seem to be skewed by the final data points, one of which appears to be a singe measurement, and there is no statistical validation for the relatively small difference. In any case, this is not a convincing way to show drug target specificity because the mutant cells have already adjusted to the absence of SHP2 in unknown ways. The only thing it shows convincingly is that 11a-1 has some off-target effects at high concentrations. I suggest dropping this panel and simply citing previous work demonstrating specificity in vitro (reference 13 in the msc).
2. The methods refer to “fully GTP loaded” RAS proteins. How is full loading assessed?
3. The modifier “highly” (text line 99) is unhelpful because there is no comparison. Please drop this word. The word “only” (text line 198) seems too strong, as there does appear to be a faint band in the Vehicle lane - please reword. Finally, “nearly abolished” seems a bit too strong for describing the large drop in sensitivity to SOS1 (line 212).

Oversights and inconsistencies
1. The abstract refers to “RAS” on line 41, but KRAS everywhere else.
2. I believe Line 106 should refer to Fig. S1g, h and i.
3. The FAK mutants used in Fig. S3 need to be described briefly somewhere.
4. Data described on lines196-198 are supposed to be in Supplemental Fig. S2, but I do not see anything there that matches the text description.

Confusing style and suggestions
1. Line 66 should refer to “spontaneous glioblastoma,” not “spontaneous mutant HRAS.”
2. Line 74 should include a brief description of “GAPs.” It would also be easier for non-experts to follow if the authors stick to GEF in the introduction, then introduce SOS1 as a specific type of GEF in the Results section.
3. The manuscript uses a slash to indicate two related proteins (H/NRAS) and also to designate signal pathways (MEK/ERK, KRAS/MAPK, others). For pathways it might be more clear if they use just the first protein (e.g., signaling downstream of RAF), a written description (MEK-to-ERK) or a text arrow (KRAS → MAPK). On line 295, try “Src- and SHP2-mediated.”
4. Text line 82 refers to “(p)KRAS,” whereas the term “pKRAS” is used everywhere else.
5. Delete Lines 247-249. These points are covered in the Discussion, where they belong.
6. Lines 293-295, using both “’dark state’” and “‘silenced’ state” is confusing. I like ‘dark state’ better, but either one used consistently could work.
7. Figure 5a is helpful but should be revised on two points. First, the nucleotide exchange would be better depicted as GDP release followed by GTP binding. I realize that it is common practice to use a single arrow showing GTP coming in followed by GDP leaving, but emphasizing that GDP comes off first should be helpful to readers. Second, the phrase “cell growth suppression” (right side below ‘Dark State’) is misleading because it implies that pKRAS-GTP sends an anti-proliferation signal, when the data suggest only that phosphorylation lowers the intensity of a positive signal.

Nomenclature
1. Please replace “c-Src” with “Src,” as it appears in the Abstract, throughout the text and figures. The “c” is not part of this gene or protein name. The “c-“ designation is only meaningful when distinguishing cellular and viral oncogenes. “c-Cbl” should also be replaced with “Cbl” (Line 127). Technically, “Src” should be SRC if you are referring to the human protein, but that is a minor point.
2. Please make clear in the Methods section whether your KRAS expression constructs encode the KRAS2A or 2B isoform (alternate C-termini).
3. I strongly recommend adopting the standard superscript nomenclature for mutant genes and proteins: KRASG12V and SHP2E76K.
4. For p120GAP, the most appropriate a.k.a. is RASA1, which is the official name in gene and protein databases that readers might consult for more information. Please use either of these rather than GAP-334.

Reviewer #3:
Remarks to the Author:
A couple of recent publications provide convincing data for and suggest the clinical evaluation of novel orally bioavailable SHP2-inhibitors not only for RTK-driven cancers but also for tumors with hyperactive (K)RAS-signaling, either due to mutations in RAS-regulatory molecules such as the RAS-GTPase activating protein NF1, due to amplification of wild-type KRAS, or even due to KRAS-mutations (Chen YN et al., Nature 2016; Refs. 6-10). However, mechanistic insight into how SHP2 regulates (mutant) KRAS-activity has remained scarce to date and is limited to hints towards a role in GTP-loading (Refs. 6+10).
This work by Kano Y et al. extends from previous data by the Ohh-Lab providing evidence that not only HRAS and NRAS (Refs. 4+5) but also KRAS is directly phosphorylated by the kinase src, being counterbalanced by the phosphatase SHP2, which directly impacts on GTP-loading, -cycling, and effector recruitment of KRAS. The authors use nuclear magnetic resonance, mass spectrometry and biolayer interferometry in mainly in vitro assays to convincingly make this very novel point regarding mechanistics introducing the terminology of a `silent/dark state` phospho-KRAS-GTP, supporting the translational claims of the aforementioned manuscripts. Thus, the data presented here is of high interest and great importance to researches in the field and informs planning of future experiments and clinical trials.

In general, the paper is very well written, clear-cut and previous literature is referenced thoroughly. The authors' approach is valid, the data, data presentation and statistical analysis appear solid, and the conclusions are appropriate, modest and seem robust.

However, the following issues should be addressed before consideration for publication in Nature Communications:

Major issues:

1. To my knowledge c-CBL is not a tyrosine kinase but a ubiquitin-ligase. I cannot follow here. Please explain and/or adjust Line 127 and Suppl. Fig. 3b.
I suppose c-CBL was used as a negative control since it is known to mediate SRC-ubiquitination and subsequent -degradation?

2. Regarding Fig. 3e,f,h an experiment with double-knockouts, or at least with a SRC-inhibitor in SHP2-/- cells would be of value to further strengthen and support the deduction that RAS-phosphorylation results in impaired ERK-phosphorylation (an to a lesser extent AKT-phosphorylation), especially since the manuscript lacks direct evidence that the phenotype observed in SHP2-/- cells is dependent on SRC.

3. Looking at Fig. 3h and 3j and the corresponding text in lines 191-200, together with Fig. 5a one wonders: Obviously the relative amount of both, mono- and dephosphorylated RAS together, is but a small fraction of the whole pool of RAS-proteins present (compared with the faster migrating band of unphosphorylated RAS). Let a very favorable estimation be around 20% of RAS proteins in the purported phosphorylated 'dark-state' in the respective experiments, then they would be outnumbered 4 over 1 by unphosphorylated RAS molecules, perfectly capable of 'canonical' signaling and, in the authors' model, refractory to SHP2-inhibition.
Thus the data in Fig. 3h+j actually suggests that other additional mechanisms contribute to the effects seen on signaling and tumor growth, respectively. This fact needs to be addressed and explained urgently, at least it is to be very openly discussed in detail. In lines 297-300 I do see a start, but especially in the KRAS-mutant context of Fig. 3j this explanation is insufficient.

4. It would be of great value if the authors could complement the experiments shown in Fig. 4b-e with the KRAS G12V mutant (as in Fig. 4g).

5. A. Quite a body of literature has reported a positive role for SRC in many aspects of tumor development, invasion and metastasis, in a variety of malignancies. In the model suggested here it acts as a tumor suppressor.
B. Importantly, SRC has been suggested to be an oncogenic co-player for mutant RAS (e.g. Poon CPC et al., Int J Mol Sci 2018), especially in pancreatic cancer (Shields DJ et al., Oncogene 2011; Morton J et al. Gastroenterology 2010). The data presented here suggests the opposite.
C. SRC-inhibition as a single agent-approach has demonstrated only minimal therapeutic activity in various types of solid tumors during early clinical trials. However, targeting SRC in cancer is still attractive for some (Zhang S and Yu D, Trends Pharmacol Sci 2012) and clinical trials with combination approaches are ongoing. Would the authors object?

> I feel like the above-mentioned items (A., B. and C.) should be addressed and discussed in order to defend and strengthen the conclusions made in the manuscript and to draw a more differentiated picture of the whole context.

Minor issues:

1. Line 52: better (suggestion): ...cancer initiation and progression...

2. The statement in lines 74-75 appears too strong since a couple of manuscripts have demonstrated dependency of (certain) KRAS-mutants on GTP-loading and RAS-GEFs like SOS (e.g. Ref. 10; Lito P et al., Science 2016; Patricelli MP et al., Cancer Discov 2016).
3. Line 106: The reference for Suppl. Fig. 1i is missing.
4. Line 109: The basis for the choice of a MEK-inhibitor as positive control is cryptic
5. Line 112: Data on mouse-weight is not provided for the whole course of treatment. (Cumulative) toxicity might appear a later point in time. The main text should be adjusted or better, Suppl. Fig. 2b updated.
6. Line 113: What about pAKT levels in the xenografts?
7. Line 131: There is no experimental evidence provided for a reversibility of the de-phosphorylation.
8. Line 150: tiny typing error > Tyr64
9. Lines 174-176: Why not mention: …pKRAS, WT and 12V,…
10. Line 191: Why not add a statement about the phosphorylation of SHP2 and src alongside RAS, since it's shown in Fig. 3g.
11. Line 262: An interesting reference that could be added for pancreatic cancer is: Zheng J et al., Nat Genetics 48, 747-757 (2016).
12. Line 270: This is a bit confusing. As stated in line 267 already there is only one single PTP domain.
13. Line 340: ...as previously generated... ?
14. Lines 460+474: Production methods or references for 15-N-KRAS and SOScat should be provided.
15. Lines 509-510: Source, production methods or references for GST-BRAF-RBD and GST-biosensors should be given.
16. Line 526: 'STR' needs to be explained.
17. Line 766: How was GDP-/GTP-loading performed? Should be added to the methods section.
18. Line 777: Suppl. Fig. 1 does not contain full spectra. Are the authors referring to Fig. 1c?
19. Fig. 3d: How were the values below the PD-blots determined? The authors should explain and indicate.
20. Fig. 4b-e: How many times were the experiments performed? How was the rate of exchange/hydrolysis fold increase determined? Error bars for Fig. 4e should be provided.
21. Suppl. Fig. 3b: Was anti-RAS or anti-HA IP performed? Is the labeling correct?

Reviewer #4:
Remarks to the Author:
Kano et al. combined biochemical, cell biological, and structural biological results of KRAS phosphorylation to decipher the molecular mechanism of the well known observation that RAS-dependent cancer treatment benefits from SHP2 inhibition. A detailed understanding of the RAS GTPase cycle is of everlasting interested because of its close relation to oncogenesis. The authors can convincingly show that c-Scr selectively phosphorylates Tyr32 and Tyr64 of KRAS in vivo and in vitro and at which step the GTPaes cycle gets altered by this PTM and subsequent SHP2 dephosphorylation. A wealth of chronologically well designed biochemical assays allowed the authors to put the observed up and down regulated effectors into a global picture of regulation in Fig. 5 together with a structural verification from previous reports. Together, I strongly recommend publication after addressing few minor concerns.

1) The oncogenic KRAS mutants G12V and G12D were part of various assays and were well described in the corresponding result section. I missed one paragraph in the discussion summarizing these observations and few general conclusions emerging from the decoded molecular regulation of KRAS concerning these prominent mutations.
2) The full NMR spectra promised in line 777 are missing in Fig. S1.

RESPONSE TO REVIEWERS

Reviewer #1 (Remarks to the Author):
In this manuscript, the authors used a range of techniques (from detailed NMR and mass spectrometry to hard-core cell biology and animal models) to unambiguously demonstrate that KRAS is phosphorylated at Tyr32 and Tyr64 by Src and dephosphorylated by SHP2. Phosphorylation appears to disrupt the KRAS GTPase cycle and signaling while dephosphorylation restores these functions. The authors also show that inhibition of SHP2 reduces oncogenic KRAS signaling. Overall, this is a clearly presented and comprehensive piece of work that advances the field of RAS biology.

I have only two suggestions (in order of relevance/feasibility):
1) Unlike the role of Src, which has been directly interrogated using NMR, the regulatory functions of SHP2 have not been subjected to a similar analysis. While the cell-biology data is strong, it would be more convincing if the NMR analysis (e.g., measurement of reaction rates) were repeated in a KRAS/Src/SHP2 context and compared with the KRAS/Src data.
Response:
We showed that Tyr32/64 phosphorylation stalls the KRAS GTPase cycle while dephosphorylation reactivates the cycle. However, as astutely raised by the reviewer, it is unclear whether SHP2 would actually dephosphorylate tyrosyl-phosphorylated KRAS in vitro, which would be necessary for restoring the KRAS GTPase cycle. To address this question, we performed NMR analysis of KRAS dephosohorylation by SHP2 and measured the rates of dephosphorylation of Tyr32 and Tyr64 (**New Fig. 3c-e**). Briefly, KRAS was phosphorylated by Src and then separated by gel filtration with phosphatase inhibitors in the running buffer to maintain full phosphorylation of KRAS. Thus, SHP2 was partially inhibited in this assay, but nevertheless effectively dephosphorylated KRAS with a rate similar to the rate of phosphorylation by the Src kinase domain (**New Fig. 3c,d**). Moreover, we utilized mass spectrometry to definitively demonstrate phosphorylation of KRAS by Src and dephosphorylation by SHP2 in the absence of phosphatase inhibitors (**New Fig. 3e**).

2) It would be nice if the authors solved at least one structure of pKRAS, pKRAS/SOS or pKRAS/GAP complexes and replaced the somewhat speculative Fig 5 by an actual data.
Response:
We agree that a crystal structure of pKRAS would be informative. However, we were unsuccessful in producing any crystals despite numerous crystallization trials of pKRAS. This is likely due, at least in part, to sample heterogeneity (i.e., a mixture of different phosphorylation states, especially after several days of incubation required for the various crystallization trials). Moreover, the resistance of pKRAS to GAP and GEF activities, together with the structural models, suggest that phosphorylation may reduce the affinity of these interactions. Thus, crystallization of pKRAS/SOS or pKRAS/GAP complexes would represent a major, perhaps improbable, challenge. For these reasons, we feel that determining these structures is beyond the scope of the present manuscript.

--
Reviewer #2 (Remarks to the Author):
The manuscript by Kano and Gebregiworgis et al. describes the tyrosine phosphorylation of KRAS by SRC and its dephosphorylation by SHP2. The authors report striking biochemical changes associate with phosphorylation, including loss of responsiveness to well-established regulators, as well as a reduced capacity to engage an effector protein in vivo. The findings have a direct bearing on efforts to understand RAS signal transduction in multiple aspects of cell biology. There are also clear implications

for the treatment of cancers that are driven by mutant RAS proteins or their upstream activators. As such, this work should be of interest to a wide readership. There are, however, several substantive and stylistic issues that should be addressed prior to a final editorial decision.

Problems that need to be addressed:
Data presentation and interpretation
1. The data in Supplementary Figure S1a are a weak argument for the specificity of the drug 11a-1. The viability curves seem to be skewed by the final data points, one of which appears to be a singe measurement, and there is no statistical validation for the relatively small difference. In any case, this is not a convincing way to show drug target specificity because the mutant cells have already adjusted to the absence of SHP2 in unknown ways. The only thing it shows convincingly is that 11a-1 has some off-target effects at high concentrations. I suggest dropping this panel and simply citing previous work demonstrating specificity in vitro (reference 13 in the msc).
Response:
Thank you for the suggestion. The noted panel has been removed and the previous work demonstrating specificity in vitro is now cited.

2. The methods refer to “fully GTP loaded” RAS proteins. How is full loading assessed?
Response:
We assume this comment pertains to line 460 (now line 519); please note that we refer to the sample as ‘fully GDP loaded’, not GTP. This was assessed by the 1H15N HSQC spectrum, in which no peaks corresponding to GTP-loaded KRAS were detectable. Since it would be difficult to detect small traces of GTP-loaded or nucleotide-free KRAS, we have removed the word ‘fully’, and changed the wording to ‘A sample of 15N-KRAS was confirmed to be GDP-loaded by collecting a 1H-15N HSQC spectrum, then incubated with a catalytic amount of recombinant Src’.

3. The modifier “highly” (text line 99) is unhelpful because there is no comparison. Please drop this word. The word “only” (text line 198) seems too strong, as there does appear to be a faint band in the Vehicle lane - please reword. Finally, “nearly abolished” seems a bit too strong for describing the large drop in sensitivity to SOS1 (line 212).
Response:
Agreed. We have removed ‘highly’ from text line 99 (now line 100). We have rephrased line 196-198 (now line 207-211); ‘Lysates of PDAC tumors treated with SHP099 or vehicle only (**Supplementary Fig. S3**) were analyzed by Mn2+-PhosTag SDS-PAGE. The slower migrating RAS band was stronger in the SHP099-treatment group (**Fig. 4f**), suggesting that the inhibition of SHP2 via SHP099 curtailed dephosphorylation of pRAS *in vivo*.’ We have also changed the wording on line 212 (now line 223-224) to read ‘while reducing the sensitivity of pKRAS to the GEF activity of the catalytic domain of SOS.’

Oversights and inconsistencies
1. The abstract refers to “RAS” on line 41, but KRAS everywhere else.
Response:
This was not an oversight, but we thank the reviewer for raising this point of potential ambiguity. We refer to ‘RAS’ when the statement is true or applicable for H/N/KRAS. We refer to ‘KRAS’ when we are making a statement about KRAS and only KRAS because (1) we did not test the other H/NRAS or (2) it remains unclear whether the statement could be extrapolated with certainty to H/NRAS. To avoid potential confusion, we have clarified the wording throughout the text, for example, from ‘RAS genes’ to ‘three RAS genes’ (line 35). Moreover, we found that pan-RAS antibody was more reliable and robust than commercially available KRAS isoform-specific antibodies for experiments involving endogenous

proteins. Thus, interpretations following endogenous experiments generally referred to 'RAS' instead of 'KRAS' (see **Fig. 4**, lines 188-211).

2. I believe Line 106 should refer to Fig. S1g, h and i.
Response:
Corrected.

3. The FAK mutants used in Fig. S3 need to be described briefly somewhere.
Response:
Done. We have defined FAK Y576/577F and FAK P712/715A as catalytically dead and catalytically active mutants, respectively, in the figure legend (now **Supplementary Fig. S4**).

4. Data described on lines 196-198 are supposed to be in Supplemental Fig. S2, but I do not see anything there that matches the text description.
Response:
Thank you for bringing the ambiguity of that figure callout to our attention. The callout to Supplementary Fig. S2 (now **Fig. S3**) refers to the PDAC PDX used in the experiment, rather than the SDS-PAGE results. This has been clarified, which also addresses the comment about the word 'only' on line 198 (now line 207); 'Lysates of PDAC tumors treated with SHP099 or vehicle only (**Supplementary Fig. S3**) were analyzed by Mn2+-PhosTag SDS-PAGE. The slower migrating RAS band was stronger in the SHP099-treatment group (**Fig. 4f**), suggesting that the inhibition of SHP2 via SHP099 curtailed dephosphorylation of pRAS *in vivo.'*

Confusing style and suggestions
1. Line 66 should refer to "spontaneous glioblastoma," not "spontaneous mutant HRAS."
Response:
Corrected.

2. Line 74 should include a brief description of "GAPs." It would also be easier for non-experts to follow if the authors stick to GEF in the introduction, then introduce SOS1 as a specific type of GEF in the Results section.
Response:
Agreed. We have added a description of GAPs on line 60, where we discuss the GTPase cycle. As suggested we now use the term GEF in the introduction, except for one instance that describes a function that is specific to SOS.

3. The manuscript uses a slash to indicate two related proteins (H/NRAS) and also to designate signal pathways (MEK/ERK, KRAS/MAPK, others). For pathways it might be more clear if they use just the first protein (e.g., signaling downstream of RAF), a written description (MEK-to-ERK) or a text arrow (KRAS → MAPK). On line 295, try "Src- and SHP2-mediated."
Response:
Thank you for this suggestion, which we have incorporated throughout the text.

4. Text line 82 refers to "(p)KRAS," whereas the term "pKRAS" is used everywhere else.
Response:
This was the first usage of the abbreviation 'p' to denote phosphorylated. To better clarify, we changed the text to read 'phosphorylated KRAS (pKRAS)'.

5. Delete Lines 247-249. These points are covered in the Discussion, where they belong.
Response:
Agreed. Done.

6. Lines 293-295, using both "'dark state'" and "'silenced' state" is confusing. I like 'dark state' better, but either one used consistently could work.
Response:
Agreed. 'Dark state' is now used throughout the text.

7. Figure 5a is helpful but should be revised on two points. First, the nucleotide exchange would be better depicted as GDP release followed by GTP binding. I realize that it is common practice to use a single arrow showing GTP coming in followed by GDP leaving, but emphasizing that GDP comes off first should be helpful to readers. Second, the phrase "cell growth suppression" (right side below 'Dark State') is misleading because it implies that pKRAS-GTP sends an anti-proliferation signal, when the data suggest only that phosphorylation lowers the intensity of a positive signal.
Response:
Thank you for these recommendations. We have revised the model in Fig. 5a (now **Fig. 7a**) regarding intrinsic and GEF-assisted nucleotide exchange (i.e., GDP release followed by GTP binding, as suggested). The phrase 'cell growth suppression' has also been changed to 'reduced signaling'.

Nomenclature
1. Please replace "c-Src" with "Src," as it appears in the Abstract, throughout the text and figures. The "c" is not part of this gene or protein name. The "c-" designation is only meaningful when distinguishing cellular and viral oncogenes. "c-Cbl" should also be replaced with "Cbl" (Line 127). Technically, "Src" should be SRC if you are referring to the human protein, but that is a minor point.
Response:
Done.

2. Please make clear in the Methods section whether your KRAS expression constructs encode the KRAS2A or 2B isoform (alternate C-termini).
Response:
Done. The expression constructs are KRAS4B, and stated in Methods.

3. I strongly recommend adopting the standard superscript nomenclature for mutant genes and proteins: $KRAS^{G12V}$ and $SHP2^{E76K}$.
Response:
Done.

4. For p120GAP, the most appropriate a.k.a. is RASA1, which is the official name in gene and protein databases that readers might consult for more information. Please use either of these rather than GAP-334.
Response:
Done. We have changed GAP334 to 'GAP domain of RASA1' throughout the text and corresponding figure legends. In the Methods, we now state 'recombinant GAP domain of RASA1 (a construct known as GAP-334)' because GAP-334 defines the exact construct.

--

Reviewer #3 (Remarks to the Author):
A couple of recent publications provide convincing data for and suggest the clinical evaluation of novel orally bioavailable SHP2-inhibitors not only for RTK-driven cancers but also for tumors with hyperactive (K)RAS-signaling, either due to mutations in RAS-regulatory molecules such as the RAS-GTPase activating protein NF1, due to amplification of wild-type KRAS, or even due to KRAS-mutations (Chen YN et al., Nature 2016; Refs. 6-10). However, mechanistic insight into how SHP2 regulates (mutant) KRAS-activity has remained scarce to date and is limited to hints towards a role in GTP-loading (Refs. 6+10).
This work by Kano Y et al. extends from previous data by the Ohh-Lab providing evidence that not only HRAS and NRAS (Refs. 4+5) but also KRAS is directly phosphorylated by the kinase src, being counterbalanced by the phosphatase SHP2, which directly impacts on GTP-loading, -cycling, and effector recruitment of KRAS. The authors use nuclear magnetic resonance, mass spectrometry and biolayer interferometry in mainly in vitro assays to convincingly make this very novel point regarding mechanistics introducing the terminology of a `silent/dark state` phospho-KRAS-GTP, supporting the translational claims of the aforementioned manuscripts. Thus, the data presented here is of high interest and great importance to researches in the field and informs planning of future experiments and clinical trials.

In general, the paper is very well written, clear-cut and previous literature is referenced thoroughly. The authors' approach is valid, the data, data presentation and statistical analysis appear solid, and the conclusions are appropriate, modest and seem robust.

However, the following issues should be addressed before consideration for publication in Nature Communications:

Major issues:
1. To my knowledge c-CBL is not a tyrosine kinase but a ubiquitin-ligase. I cannot follow here. Please explain and/or adjust Line 127 and Suppl. Fig. 3b. I suppose c-CBL was used as a negative control since it is known to mediate SRC-ubiquitination and subsequent degradation?
Response:
We apologize for the lack of explanation. Yes, we used CBL as a negative control as it is known to target Src for ubiquitin-mediated destruction. We have clarified this information in the text. Furthermore, we have examined SYK, another tyrosine kinase in addition to FAK, which also failed to phosphorylate KRAS (**New Supplementary Fig. S4b**).

2. Regarding Fig. 3e,f,h an experiment with double-knockouts, or at least with a SRC-inhibitor in SHP2-/- cells would be of value to further strengthen and support the deduction that RAS-phosphorylation results in impaired ERK-phosphorylation (and to a lesser extent AKT-phosphorylation), especially since the manuscript lacks direct evidence that the phenotype observed in SHP2-/- cells is dependent on SRC.
Response:
We agree with this astute comment. However, considering that both SHP2 and Src have multiple targets, we experimentally addressed the above comment with the understanding that a clear interpretation from such an experiment may be difficult. We treated SYF-/- and SHP2-/- MEFs with a non-ATP competitive substrate pocket-directed Src inhibitor KX2-391 and observed that while there was no noticeable change to pERK level in SYF-/- MEFs, which was expected, there was a modest increase in pERK levels in SHP2-/- MEFs in the absence and presence of PDGF-BB stimulation (**New Supplementary Fig. S6a**). Consistent with this observation, siRNA-mediated knockdown of Src in SHP2-/- MEFs slightly increased the level of pERK (**New Supplementary Fig. S6b**). These results support the notion that the attenuation of RAS-to-ERK signaling in SHP2-/- cells is dependent, at least in part, to Src.

3. Looking at Fig. 3h and 3j and the corresponding text in lines 191-200, together with Fig. 5a one wonders: Obviously the relative amount of both, mono- and dephosphorylated RAS together, is but a small fraction of the whole pool of RAS-proteins present (compared with the faster migrating band of unphosphorylated RAS). Let a very favorable estimation be around 20% of RAS proteins in the purported phosphorylated 'dark-state' in the respective experiments, then they would be outnumbered 4 over 1 by unphosphorylated RAS molecules, perfectly capable of 'canonical' signaling and, in the authors' model, refractory to SHP2-inhibition. Thus the data in Fig. 3h+j actually suggests that other additional mechanisms contribute to the effects seen on signaling and tumor growth, respectively. This fact needs to be addressed and explained urgently, at least it is to be very openly discussed in detail. In lines 297-300 I do see a start, but especially in the KRAS-mutant context of Fig. 3j this explanation is insufficient.

Response:

This is an interesting comment raised by the reviewer, which continues to generate thoughtful discussions in the signaling field. As suggested, we have now discussed this question openly in the text. Firstly, it is important to underscore that the nature of RAS phosphorylation and GTPase cycle is both dynamic and transient. Considering that biochemical and biophysical assays generally measure a 'snap shot' activity at a given moment in time, it is therefore difficult to ascertain precisely how much of the total pool of RAS is actually engaged or required to activate downstream signaling cascade to cause a biologically meaningful change. This also raises the second critical point, which is that RAS binds to numerous effector molecules to promote various cellular processes ranging from actin cytoskeletal integrity, cell polarity to cell proliferation. Thus, it is thought that not all RAS would be required or engaged to exact a specific cellular phenotypic change. Related to this notion is the concept of threshold. That is, a signaling cascade would cease to be activated if and when an activating event (i.e., specific posttranslational modification) falls below the necessary threshold. For example, if a given pathway activation requires a minimum 90% of the total unmodified canonical RAS GTPase then, hypothetically, phosphorylation of 20% of RAS would be sufficient to cease the signaling output. Thirdly, spatiotemporal coordination of the 'minor' fraction of KRAS at a given moment in time would have a significant impact on downstream signaling output. Notably, the activation of receptor tyrosine kinases upon ligand interaction would be coordinated with activated SOS, which are thus poised to act on the relatively minor pool of KRAS that is, however, relevant to signaling. Thus, phosphorylation-mediated regulation of this spatiotemporally relevant population of KRAS would be predicted to have a significant impact on signaling.

Regarding the comment that there may be additional mechanisms contributing to effects seen on signaling and tumor growth, we acknowledge that this is likely to be true as SHP2 has several other targets and non-catalytic roles. For example, SHP2 has been implicated in regulating GEF and GAP localization by dephosphorylating the p120 RASGAP docking sites on EGFR and GAB1, or GRB2 binding sites on Sprouty, thereby reversing its negative regulation of SOS recruitment. In other words, maintaining or prolonging KRAS phosphorylation is not the only mechanism of action of SHP2 inhibitors. Thus, we suggest conservatively that the *in vivo* animal model study supports 'the potential utility of SHP2 inhibitors in the treatment of mutant KRAS-driven PDAC'.

4. It would be of great value if the authors could complement the experiments shown in Fig. 4b-e with the KRAS G12V mutant (as in Fig. 4g).

Response:

Thank you for this suggestion. We analyzed KRAS G12V mutant and consistent with the previously reported findings by Hunter et al[1], we showed that this particular mutant has a very slow GTP hydrolysis rate, which was not increased by GAP (**New Supplementary Fig. S7**). Furthermore, we show that phosphorylation of KRAS G12V has negligible impact on hydrolysis and nucleotide exchange rates, but it

attenuates the sensitivity to SOS (**New Supplementary Fig. S7**). These results suggest that KRAS G12V mutant conforms to our dark state model whereby phosphorylation decouples its GTPase cycle from regulation (even though it is already insensitive to GAP) and impairs its interaction with effector RAF (**Fig. 6c, d**).

5. A. Quite a body of literature has reported a positive role for SRC in many aspects of tumor development, invasion and metastasis, in a variety of malignancies. In the model suggested here it acts as a tumor suppressor.
B. Importantly, SRC has been suggested to be an oncogenic co-player for mutant RAS (e.g. Poon CPC et al., Int J Mol Sci 2018), especially in pancreatic cancer (Shields DJ et al., Oncogene 2011; Morton J et al. Gastroenterology 2010). The data presented here suggests the opposite.
C. SRC-inhibition as a single agent-approach has demonstrated only minimal therapeutic activity in various types of solid tumors during early clinical trials. However, targeting SRC in cancer is still attractive for some (Zhang S and Yu D, Trends Pharmacol Sci 2012) and clinical trials with combination approaches are ongoing. Would the authors object?
> I feel like the above-mentioned items (A., B. and C.) should be addressed and discussed in order to defend and strengthen the conclusions made in the manuscript and to draw a more differentiated picture of the whole context.
Response:
Thank you for raising these points, which we have addressed and discussed in the text. Similar to RAS, Src has been shown to play an important role in tumor development and therefore targeting Src was anticipated and continues to be attractive as an anti-cancer strategy. However, a major challenging issue in the clinical development of Src inhibitors has been the lack of effective response biomarkers to guide the design of clinical trials, which is partly due to the enormous complexity of Src signaling. Notably, all the completed clinical trials of Src inhibitors were performed in unselected patients, and despite the promise, Src inhibitor therapy on several types of metastatic solid tumors including breast, prostate, head and neck, colon, non small cell lung, and pancreatic cancers has shown no significant clinical benefit as a single agent in Phase II trials[2]. In a genetically engineered mouse model of PDAC, Src inhibitor dasatinib, which also targets ABL, had negligible effect on tumor growth or survival, although it reduced the incidence of metastasis[3]. While there have also been some promising reports for Src-targeted therapy[2] and clinical trials with combination approaches are ongoing, these results underscore the complexity and difficulty of targeting Src to achieve significant clinical benefit. Pre-clinical data suggest that cells with elevated Src activity are more likely to respond to Src inhibition, while tumors with diminished Src signaling resulting from alternative oncogenic pathways may contribute to *de novo* resistance to Src inhibitors[4-7]. These, albeit limited, studies demonstrate that hyperactive Src signaling may potentially serve as a biomarker for successful targeting of Src and clinical efficacy. However, other molecular alterations in cancers may impact on the response of cancer cells to Src inhibitors. For example, c-MET amplification in gastric cancer as well as the autophagy pathway were shown to promote resistance to Src inhibitors[7,8] while acquired resistance of ER+ breast cancers to the Src family kinase inhibitor saracatinib is associated with the reactivation of the mTOR pathway[9,10]. These results suggest that cancer cells can acquire resistance to Src inhibitor via multiple genetic alterations. Thus, further clinical studies are needed to develop more reliable biomarkers that can guide clinical trials. To our knowledge, KRAS mutational status has not been evaluated as a biomarker for response to Src inhibitors. In light of our present study suggesting tumor suppressive role of Src in the context of oncogenic KRAS as well as recent reports demonstrating clinical utility of SHP2 inhibitors in the management of mutant KRAS-driven cancers[11-15], inclusion of KRAS mutational status would be prudent for future clinical trials of Src inhibitors as mono or combination therapies.

Minor issues:
1. Line 52: better (suggestion): ...cancer initiation and progression...
Response:
Agreed. Done.

2. The statement in lines 74-75 appears too strong since a couple of manuscripts have demonstrated dependency of (certain) KRAS-mutants on GTP-loading and RAS-GEFs like SOS (e.g. Ref. 10; Lito P et al., Science 2016; Patricelli MP et al., Cancer Discov 2016).
Response:
This is an astute comment. We have accordingly revised the statement from 'oncogenic KRAS mutations confer resistance to GAPs and remain highly GTP loaded without requiring activation by SOS' to 'oncogenic KRAS mutations confer resistance to GAPs and most would therefore exhibit elevated GTP loading even in the absence of SOS activity'. As the reviewer aptly points out certain KRAS mutants do require GEF activity, e.g., G12C, which is the subject of the above noted reports. KRAS G12C is a special case because its intrinsic hydrolysis activity is near wild-type[1], thus it depends upon GEF activity for reactivation.

3. Line 106: The reference for Suppl. Fig. 1i is missing.
Response:
Corrected (now Line 107: **Supplementary Fig. S1f,g** and **h**).

4. Line 109: The basis for the choice of a MEK-inhibitor as positive control is cryptic
Response:
We previously determined that Trametinib, a MEK inhibitor, has a significant anti-tumor effect in a similar PDX model (**New Supplementary Fig. S2c**). We have added this explanation to the main text.

5. Line 112: Data on mouse-weight is not provided for the whole course of treatment. (Cumulative) toxicity might appear at later point in time. The main text should be adjusted or better, Suppl. Fig. 2b updated.
Response:
Done. We first determined maximum tolerable dose of SHP099 in SCID mice and observed no discernable weight loss in 100 mg/kg treatment group while 150 mg/kg treatment showed slight weight loss and 200 and 250 mg/kg treatments were intolerable (**New Supplementary Fig. S2a** and **b**). **Supplementary Fig. S3b** (previously Fig. S2b) shows the weight of mice over the whole course or 'duration of treatment'. We have added 'Treatment Start' at the beginning of the graph to avoid potential ambiguity.

6. Line 113: What about pAKT levels in the xenografts?
Response:
Thank you for the suggestion. We have analyzed the tumor xenografts and added pAKT and AKT data, which showed that SHP099 treatment was associated with reduced level of pAKT, similar to pERK and pMEK (**Supplementary Fig. S3c**).

7. Line 131: There is no experimental evidence provided for a reversibility of the de-phosphorylation.
Response:
Thank you for pointing out this ambiguous statement, which was intended to mean that phosphorylation is reversible. To avoid ambiguity, we have removed the word 'reversibly'.

8. Line 150: tiny typing error > Tyr64
Response:
Corrected.

9. Lines 174-176: Why not mention: …pKRAS WT and 12V,…
Response:
Done.

10. Line 191: Why not add a statement about the phosphorylation of SHP2 and src alongside RAS, since it's shown in Fig. 3g.
Response:
Agreed. We have added SHP2 and Src to the statement (now Line 201-202).

11. Line 262: An interesting reference that could be added for pancreatic cancer is: Zheng J et al., Nat Genetics 48, 747-757 (2016).
Response:
Thank you for this suggestion. We have added the noted reference.

12. Line 270: This is a bit confusing. As stated in line 267 already there is only one single PTP domain.
Response:
Thank you for pointing out this typo. We have changed PTP domains to domain.

13. Line 340: ...as previously generated... ?
Response:
Changed to 'generated as previously described'.

14. Lines 460+474: Production methods or references for 15-N-KRAS and SOScat should be provided.
Response:
Done. The expression and purification of isotopically labeled (15N) KRAS and SOScat are described with references (lines 504-508 and 487-491).

15. Lines 509-510: Source, production methods or references for GST-BRAF-RBD and GST-biosensors should be given.
Response:
Done. Requested information has now been added.

16. Line 526: 'STR' needs to be explained.
Response:
Done. Revised to 'short tandem repeat (STR) profiling'.

17. Line 766: How was GDP-/GTP-loading performed? Should be added to the methods section.
Response:
Done. WT KRAS was purified from E. Coli in the GDP bound form, while G12V purifies as a mixture of GDP- and GTP-bound. The KRAS G12V was thus incubated to allow hydrolysis of GTP and it was confirmed by collecting a 1H 15N HSQC spectrum that it was GDP loaded before we used the sample for further experimentation. This has been added to the Methods (lines 519-523). We loaded KRAS with GTP by incubating it with a 10-fold excess of GTP in the presence of EDTA, which were then removed by gel filtration chromatography. This is described in the Methods with reference (lines 536-539).

18. Line 777: Suppl. Fig. 1 does not contain full spectra. Are the authors referring to Fig. 1c?
Response:
We apologize for this error, which is now corrected. The full spectrum is indeed shown in **Fig. 1c**.

19. Fig. 3d: How were the values below the PD-blots determined? The authors should explain and indicate.
Response:
We apologize for this oversight. We have added an explanation for the quantification of visualized bands in now **Fig. 6c** and **d**; 'Numerical values indicate the ratio of quantified signals over the control (lane 1 of each panel; set at 1.0) determined by densitometry using ImageJ software.'

20. Fig. 4b-e: How many times were the experiments performed? How was the rate of exchange/hydrolysis fold increase determined? Error bars for Fig. 4e should be provided.
Response:
Each experiment was conducted at least twice in duplicates. The rates of the exchange/hydrolysis were derived from fitting the curves (obtained from 1H-15N HSQC peaks) to a one-phase exponential decay function. The pooled mean rates were then used to calculate the fold changes for each sample upon addition of GAP or GEF (e.g., the fold change due to SOScat addition = mean of the rate of SOScat catalyzed exchange / mean of the rate of the intrinsic exchange). The error bars in **Fig. 5c** and **5e** indicate the range of the fold changes. We have added a statement on how these data were plotted in the corresponding figure legend. Error bars have been added to now **Fig. 5e**.

21. Suppl. Fig. 3b: Was anti-RAS or anti-HA IP performed? Is the labeling correct?
Response:
The labeling is correct. FAK mutants, Cbl and KRAS have HA-tag. Thus, we performed IP with an anti-RAS antibody to immunoprecipitate HA-KRAS for this particular experiment (now **Suppl. Fig. S4b**).

--
Reviewer #4 (Remarks to the Author):
Kano et al. combined biochemical, cell biological, and structural biological results of KRAS phosphorylation to decipher the molecular mechanism of the well known observation that RAS-dependent cancer treatment benefits from SHP2 inhibition. A detailed understanding of the RAS GTPase cycle is of everlasting interested because of its close relation to oncogenesis. The authors can convincingly show that c-Scr selectively phosphorylates Tyr32 and Tyr64 of KRAS in vivo and in vitro and at which step the GTPaes cycle gets altered by this PTM and subsequent SHP2 dephosphorylation. A wealth of chronologically well designed biochemical assays allowed the authors to put the observed up and down regulated effectors into a global picture of regulation in Fig. 5 together with a structural verification from previous reports. Together, I strongly recommend publication after addressing few minor concerns.

1) The oncogenic KRAS mutants G12V and G12D were part of various assays and were well described in the corresponding result section. I missed one paragraph in the discussion summarizing these observations and few general conclusions emerging from the decoded molecular regulation of KRAS concerning these prominent mutations.
Response:

We agree with the reviewer and have included a summarizing statement in the discussion regarding the KRAS mutants (lines 308-312); ‘Importantly, KRAS mutations frequently occur at codon 12 and substitution mutations encoding G12D and G12V are amongst the most prevalent in PDAC tumors. Notably, we show that Src- and SHP2-mediated regulation of KRAS extends to these common KRAS mutants and that the inhibition of SHP2 activity disrupts the phosphorylation cycle, shifting the equilibrium of the oncogenic KRAS towards the ‘dark state’ (**Fig. 7a**).’

2) The full NMR spectra promised in line 777 are missing in Fig. S1.

Response:

We apologize for this oversight, which is now corrected. The full spectrum is shown in **Fig. 1c**.

--

References

1 Hunter, J. C. *et al.* Biochemical and Structural Analysis of Common Cancer-Associated KRAS Mutations. *Molecular cancer research : MCR* **13**, 1325-1335, doi:10.1158/1541-7786.mcr-15-0203 (2015).

2 Zhang, S. & Yu, D. Targeting Src family kinases in anti-cancer therapies: turning promise into triumph. *Trends in pharmacological sciences* **33**, 122-128, doi:10.1016/j.tips.2011.11.002 (2012).

3 Morton, J. P. *et al.* Dasatinib inhibits the development of metastases in a mouse model of pancreatic ductal adenocarcinoma. *Gastroenterology* **139**, 292-303, doi:10.1053/j.gastro.2010.03.034 (2010).

4 Nagaraj, N. S., Smith, J. J., Revetta, F., Washington, M. K. & Merchant, N. B. Targeted inhibition of SRC kinase signaling attenuates pancreatic tumorigenesis. *Molecular cancer therapeutics* **9**, 2322-2332, doi:10.1158/1535-7163.mct-09-1212 (2010).

5 Arcaroli, J. J. *et al.* Gene array and fluorescence in situ hybridization biomarkers of activity of saracatinib (AZD0530), a Src inhibitor, in a preclinical model of colorectal cancer. *Clinical cancer research : an official journal of the American Association for Cancer Research* **16**, 4165-4177, doi:10.1158/1078-0432.ccr-10-0066 (2010).

6 Suwaki, N. *et al.* A HIF-regulated VHL-PTP1B-Src signaling axis identifies a therapeutic target in renal cell carcinoma. *Science translational medicine* **3**, 85ra47, doi:10.1126/scitranslmed.3002004 (2011).

7 Okamoto, W. *et al.* Identification of c-Src as a potential therapeutic target for gastric cancer and of MET activation as a cause of resistance to c-Src inhibition. *Molecular cancer therapeutics* **9**, 1188-1197, doi:10.1158/1535-7163.mct-10-0002 (2010).

8 Ahn, J. H. & Lee, M. Suppression of autophagy sensitizes multidrug resistant cells towards Src tyrosine kinase specific inhibitor PP2. *Cancer letters* **310**, 188-197, doi:10.1016/j.canlet.2011.06.034 (2011).

9 Chen, Y. *et al.* Combined Src and ER blockade impairs human breast cancer proliferation in vitro and in vivo. *Breast cancer research and treatment* **128**, 69-78, doi:10.1007/s10549-010-1024-7 (2011).

10 Chen, Y. *et al.* Combined Src and aromatase inhibition impairs human breast cancer growth in vivo and bypass pathways are activated in AZD0530-resistant tumors. *Clinical cancer research : an official journal of the American Association for Cancer Research* **15**, 3396-3405, doi:10.1158/1078-0432.ccr-08-3127 (2009).
11 Wong, G. S. *et al.* Targeting wild-type KRAS-amplified gastroesophageal cancer through combined MEK and SHP2 inhibition. *Nature medicine* **24**, 968-977, doi:10.1038/s41591-018-0022-x (2018).
12 Ruess, D. A. *et al.* Mutant KRAS-driven cancers depend on PTPN11/SHP2 phosphatase. *Nature medicine* **24**, 954-960, doi:10.1038/s41591-018-0024-8 (2018).
13 Nichols, R. J. *et al.* RAS nucleotide cycling underlies the SHP2 phosphatase dependence of mutant BRAF-, NF1- and RAS-driven cancers. *Nature cell biology* **20**, 1064-1073, doi:10.1038/s41556-018-0169-1 (2018).
14 Mainardi, S. *et al.* SHP2 is required for growth of KRAS-mutant non-small-cell lung cancer in vivo. *Nature medicine* **24**, 961-967, doi:10.1038/s41591-018-0023-9 (2018).
15 Fedele, C. *et al.* SHP2 Inhibition Prevents Adaptive Resistance to MEK Inhibitors in Multiple Cancer Models. *Cancer discovery* **8**, 1237-1249, doi:10.1158/2159-8290.cd-18-0444 (2018).

Reviewers' Comments:

Reviewer #2:
Remarks to the Author:
The authors' revisions have adequately addressed all concerns.
John Colicelli

Reviewer #3:
Remarks to the Author:
The authors have responded to our concerns with new data and textual changes satisfactorily. The manuscript has been improved significantly and the data are now very convincing. I have no further suggestions.
